# Effect of 2 Clinical Decision Support Strategies on Chronic Kidney Disease Outcomes in Primary Care: A Cluster Randomized Trial

**DOI:** 10.1001/jamanetworkopen.2018.3377

**Published:** 2018-10-26

**Authors:** Jennifer K. Carroll, Gerald Pulver, L. Miriam Dickinson, Wilson D. Pace, Joseph A. Vassalotti, Kim S. Kimminau, Brian K. Manning, Elizabeth W. Staton, Chester H. Fox

**Affiliations:** 1 Department of Family Medicine, University of Colorado Denver, Aurora; 2 American Academy of Family Physicians, Leawood, Kansas; 3 DARTNet Institute, Inc, Aurora, Colorado; 4 National Kidney Foundation, New York, New York; 5 Icahn School of Medicine at Mount Sinai, New York, New York; 6 Department of Family Medicine, University of Kansas Medical Center, Kansas City; 7 Greater Buffalo Accountable Healthcare Network, Buffalo, New York

## Abstract

**Importance:**

Information is needed about optimal strategies to improve evidence-based treatment of chronic kidney disease (CKD) in primary care.

**Objective:**

To determine whether a multimodal intervention delays annualized loss of estimated glomerular filtration rate (eGFR) in stages 3 and 4 CKD.

**Design, Setting, and Participants:**

This pragmatic cluster randomized clinical trial enrolled 42 primary care practices located in nonhospital settings with electronic health record systems. Practices were recruited through the American Academy of Family Physicians National Research Network. The study was conducted January 2013 through January 2016.

**Interventions:**

Practices were randomized at the organization level to either the clinical decision support (CDS) plus practice facilitation (PF) group (n = 25) or CDS group (n = 17) using covariate constrained randomization. Both groups received point-of-care CDS to prompt screening, diagnosis, and treatment of CKD; the intervention group also received PF based on the 9-point TRANSLATE model (target, use point-of-care reminder systems, get administrative buy-in, network information systems using registries, site coordination, local physician champion, audit and feedback, team approach, and education).

**Main Outcomes and Measures:**

The primary outcome measure was eGFR over time. Secondary outcome measures were systolic blood pressure over time, change in hemoglobin A_1c_ (HbA_1c_) over time, avoidance of nonsteroidal anti-inflammatory medications, use of angiotensin converting enzyme inhibitor or angiotensin-renin blocker medication, early recognition and diagnosis of CKD, blood pressure control, and smoking cessation.

**Results:**

In this cluster randomized trial of 30 primary care practices comprising 6699 patients, there were 1685 patients in the control group (10 practices) and 5014 patients in the intervention group (20 practices). The final sample of practices differed from the original set of randomized practices owing to dropout. Patients in the practices were similar at baseline for age (mean [SD], 71.3 [9.6] years), sex (2716 male [40.5%]), and eGFR. There was a significant difference in eGFR slopes for patients in the intervention vs control group practices. The mean (SE) annualized loss of eGFR was 0.95 (0.19) in the control group in propensity-adjusted longitudinal analyses and 0.01 (0.12) in the intervention group (mean [SE] difference in slopes, 0.93 [0.23]; *P* < .001). Among patients with HbA_1c_ measures, slopes differed significantly for patients in intervention vs control practices, with a mean (SE) annualized increase of 0.14 (0.03) in HbA_1c_ for patients in control practices and a mean (SE) decline of 0.009 (0.02) for patients in intervention practices. There was a significant difference in HbA_1c_ slopes for patients in the intervention compared with control group practices (control vs intervention, −0.14; *P* < .001), but no difference in the other secondary outcomes.

**Conclusions and Relevance:**

A multimodal intervention in primary care, based on the TRANSLATE model, slowed annualized loss of eGFR. This study had several important strengths, weaknesses, and lessons learned regarding the implementation of pragmatic interventions in primary care to improve CKD outcomes.

**Trial Registration:**

ClinicalTrials.gov Identifier: NCT01767883

## Key Points

**Question:**

Can clinical decision support plus practice facilitation improve treatment of chronic kidney disease (CKD) in primary care, with the goal of delaying progression of CKD from stages 3 and 4?

**Findings:**

This cluster randomized clinical trial of 30 primary care practices comprising 6699 patients showed a significant effect in the intervention group compared with the control group in slowing the annualized loss of estimated glomerular filtration rate in patients with stages 3 and 4 CKD. There was significant improvement in hemoglobin A_1c_ over time among patients in the intervention group compared with patients in the control group, with an imbalance between groups resulting from practice dropout posing a significant limitation.

**Meaning:**

Findings suggest that a multimodal intervention in primary care can slow the progression of stages 3 and 4 CKD.

## Teaser

This cluster randomized clinical trial compares clinical decision support with vs without practice facilitation to improve treatment of chronic kidney disease in primary care as measured by annualized loss of estimated glomerular filtration rate.

## Introduction

Chronic kidney disease (CKD) is a large and growing concern in the United States, with an estimated prevalence of 15%.^1^ In 2012, the Medicare cost for CKD treatment was $87 billion, with $28.6 billion for dialysis treatment.^2,3^ Chronic kidney disease is associated with a 5-year all-cause mortality rate of 24.3% for stage 3 and 45.7% for stage 4 and a 5-year need for renal replacement of 1.3% for stage 3 and 19.9% for stage 4.^4^

This condition is poorly identified and treated in primary care. Primary care clinicians have limited knowledge of current evidence-based recommendations,^5,6,7^ such as the use of angiotensin converting enzyme inhibitor (ACEi) or angiotensin receptor blocker (ARB) therapy, avoidance of nonsteroidal anti-inflammatory drugs (NSAIDs), control of blood glucose and blood pressure, and smoking cessation. Because CKD is underrecognized and undertreated in primary care offices, the implementation of evidence-based interventions is low.^5,6^

The Chronic Care Model has been widely accepted and used in primary care practices as a means to improve evidence-based care.^8,9^ For implementing the Chronic Care Model, Peterson et al^10^ developed the 9-point TRANSLATE action plan (set your target, use point-of-care reminder systems, get administrative buy-in, network information systems using registries, site coordination, local physician champion, audit and feedback, team approach, and education), including clinical decision support (CDS) and practice facilitation. This model has been used successfully in diabetes care.^10^

The use of practice facilitation,^11^ point-of-care clinical decision support,^12,13^ and academic detailing^14^ show promise to help improve quality of care. Pilot studies have demonstrated improvements in recognition and treatment of CKD^15^ and maintenance of improvement over 2 years.^16^ However, to our knowledge, there have been no large pragmatic randomized clinical trials examining a multimodal approach toward improving evidence-based CKD care and associated patient outcomes in the United States.

The objective of this study was to determine whether a multimodal intervention consisting of clinical decision support (CDS) plus practice facilitation (PF) delays annualized loss of estimated glomerular filtration rate (eGFR) in CKD (defined as eGFR <60 mL/min/1.73 m^2^) compared with a control group that received CDS alone. The rationale for designing a study to slow progression of CKD was based on the need to enhance uptake of evidence-based guidelines, demonstrate improvement in relevant related outcomes (eg, glycemic control), and, if successful, achieve the triple aim of enhancing patient experience, improving population health, and reducing costs. Cluster randomization was chosen because the intervention was at the practice level and it allowed us to prevent contamination by preference by physicians or practices (selection bias).^17^ We hypothesized that patients with stage 3 and 4 CKD in the CDS plus PF group would have slower annualized loss of eGFR than patients in the CDS-only group.

## Methods

The study was conducted at 42 practice sites from January 2013 through January 2016. The protocol was approved by the institutional review boards at the State University of New York at Buffalo and the American Academy of Family Physicians National Research Network and has been previously published.^18^ The trial protocol is available in Supplement 1. Informed consent was obtained from each practice’s coordinator and lead physician prior to randomization. Patient informed consent was waived as the intervention was at the practice level. Patient data from the electronic health records (EHRs) consisted of a limited data set, and no identifiable data were shared outside the practice. This report follows the Consolidated Standards of Reporting Trials (CONSORT) reporting guideline extension to cluster randomized trials.^17^

### Design 

The study design was a parallel-group multisite cluster randomized trial (n = 42 clusters). A cluster was defined as an ambulatory primary care practice with a distinct office location.

### Participants 

Eligible practices provided ambulatory primary care as their principal function, were located in nonhospital settings, employed at least 1 primary care physician, and saw a minimum of 2000 patients in the year prior to study enrollment. All eligible patients at the practice received the study protocol assigned to their practice based on the practice’s randomized group assignment. The patient population within each practice consisted of patients with stages 3 or 4 CKD (defined as having at least 2 consecutive eGFR measurements <60 mL/min/1.73 m^2^ and >15 mL/min/1.73 m^2^ at least 3 months apart prior to baseline). Baseline eGFR (designated time 0) was the average of the last 2 eGFR measurements prior to baseline (from the 24 months before baseline). The follow-up period spanned 24 months after baseline. To be included in the study cohort, patients had to have at least 2 eGFR measurements during the 24 months prior to baseline and at least 1 eGFR measurement during the follow-up period. Exclusions were current hemodialysis or functioning kidney transplant.

### Recruitment of Practices 

Practice recruitment occurred through the American Academy of Family Physicians National Research Network and the DARTNet Institute. The American Academy of Family Physicians National Research Network is the largest practice-based research network in the United States, including more than 3000 primary care clinician members and 1300 primary care offices (including small private offices, large health systems, federally qualified health centers, and residencies). The DARTNet Institute is a nonprofit research institute that coordinates and supports research, quality improvement, and safety activities across multiple research networks through the reuse and improved collection of electronic health data.

### Interventions 

Both the CDS group and the CDS plus PF group implemented the first 4 elements of the TRANSLATE method: target, use point-of-care reminder systems, get administrative buy-in, and network information systems creating a practice population–based registry. The remaining elements, site coordination, local physician champion, audit and feedback, team approach, and education, were implemented in the CDS plus PF group only. See eTable 1 in Supplement 2 for further details.

### Control (CDS-Only) Group

The CDS-only sites had CKD decision support algorithms added to their EHR systems. They received academic detailing and technical support to learn how to view and use the algorithms and a quick reference guide for the treatment of CKD.

The key performance measures and their targets were control of blood pressure, glucose, and low-density lipoprotein (LDL) cholesterol; use of ACEi/ARB; smoking cessation; and avoidance of NSAID or cyclooxygenase 2 inhibitor medications.

### Outcomes 

The primary outcome was the annualized loss of eGFR. Secondary outcomes were change in systolic blood pressure (SBP) over time, avoidance of NSAIDs, use of ACEi and ARBs, and CKD diagnosis. Drug usage information, including ACEi, ARB, and NSAID, was drawn from prescriptions, reconciliation and history, and fulfillment information. Avoidance of NSAIDs meant that the patient’s medical record, as of the final date of the study, included no mention of NSAID usage. The HbA_1c_ value given for each patient was the most recent value recorded within the preceding year. Systolic and diastolic blood pressure are the average of the most recent (up to) 3 observations. Diagnosis of CKD was defined as no more than 1 eGFR measurement less than 15 mL/min/1.73 m^2^ and at least 1 of the following: (1) at least 1 eGFR measurement in target range (≥15 and <60 mL/min/1.73 m^2^), (2) at least 2 eGFR measurements ≥15 and <60 mL/min/1.73 m^2^ at least 90 days apart, with no value ≥60 mL/min/1.73 m^2^ between them, and (3) diagnosis of CKD by *International Classification of Diseases, Ninth Revision, Clinical Modification*, *International Classification of Diseases, Tenth Revision, Clinical Modification*, or *Systematized Nomenclature of Medicine—Clinical Terms* codes AND at least 1 eGFR measurement ≥15 and <60 mL/min/1.73 m^2^.

Sample size was based on a very conservative estimate of minimum patients per practice. With 20 practices per group and a minimum of 200 patients per practice (smallest practice estimated at 2000 patients with a conservative 10% prevalence of CKD over the life of the study), we expected a minimum of 4000 patients per group. The actual sample size was more than 14 000 patients per group. A sample size of 4000 per group provides greater than 80% power to detect a 0.17 effect size difference between 2 groups at a single time point if the intraclass correlation is 3%. This effect size was assumed based on previous results from the diabetes TRANSLATE study.^10^ In terms of change over time, a sample size of 4000 provides greater than 80% power to detect a small linear trend effect (increasing from 0 at baseline to 0.2 SD at final follow-up) with 4 observations per person and an intraclass correlation of 3%, with a random-effects structure with random intercept and random slope and 5% attrition over time.^10^ If the intraclass correlation is higher (eg, 10%) and attrition is higher (eg, 20%) the study has power to detect a medium linear trend effect (increasing from 0 at baseline up to 0.5 SD difference at final follow-up) with 4 observations per person.^19^

### Randomization

Covariate constrained randomization procedures were used to randomize clusters,^18,20^ with practices in the same organization constrained to be in the same study group if there was clinician overlap. All eligible patients within a practice were assigned to the study group designated for their practice. Prestudy data on practices were obtained from the EHR and aggregated to the practice level. Variables included baseline performance characteristics related to CKD and practice and patient panel characteristics (number of full-time equivalent physicians, mean HbA_1c_, percentage of diabetic patients, percentage with stage 4 CKD, percentage of diabetic patients with HbA_1c_ >9% (to convert HbA_1c_ to proportion of total hemoglobin, multiply by 0.01), mean eGFR, mean SBP, percentage with SBP >130 mm Hg, percentage with SBP >140 mm Hg, percentage African American, percentage Hispanic/Latino, percentage uninsured). Stratification variables included geographic region and organization. Randomization occurred in 2 waves. Within each wave, all possible combinations of eligible practices in 2 groups of equal size were generated using the Interactive Matrix Language procedure in SAS statistical software, version 9.4 (SAS Institute Inc). Randomizations that were balanced on stratification variables were retained, and a balance criterion (defined as the sum of squared differences on standardized variables between control and intervention groups) was computed. After examining the distribution of the balance criterion, we established a maximum allowable difference between the groups (approximately the best 10%) and identified an optimal set of randomizations. From this set, 1 was chosen using a random number generator (SAS), and practices were assigned to the treatment group or the control group by the project biostatistician (L.M.D.). For randomization, practices were assigned a sequential identifier that was not linked to practice name until randomization was completed. There was no allocation concealment during the study. There was no blinding of practices or the research team after randomization. Practices within the same organization were grouped as a single practice for randomization because of the potential for contamination, resulting in unequal numbers of actual practice sites randomized to the 2 groups.

### Data Extraction

The DARTNet Institute extracted EHR data from sites to provide feedback reports and for the overall metrics needed to conduct evaluation. The following EHR data were extracted: identities of clinicians, care sites, and organizations; dates of visits; patient sex and year of birth; diagnoses, both current and past conditions; medication exposures, both prescriptions written and historical data obtained through reconciliation; and laboratory results, height, weight, blood pressure, and smoking status and history. The data sources are shown in eFigure 1 in Supplement 2. The source EHRs are listed in eTable 2 in Supplement 2.

For purposes of standardization, all data were converted into Observational Medical Outcomes Partnership concepts.^21^ This is a data model with standard vocabularies, developed to support transforming, characterizing, and analyzing disparate data sources across health care delivery.

### Statistical Analysis

Descriptive statistics (means, standard deviations, and percentages) were generated for patient sociodemographic measures, clinical measures, and process-of-care measures. Bivariate relationships were examined using χ^2^ tests and *t* tests.

The primary outcome for this study was eGFR over time with repeated patient measurements. Baseline was defined as the patient’s last eGFR prior to each practice’s study initiation. Multilevel modeling (general linear mixed models that are both longitudinal and hierarchical, with random coefficients for patients [intercepts and slopes] and practice random intercepts) using all available data and adjusted for covariates associated with loss to follow-up (assuming nonignorable missingness) was used for analysis (SAS PROC MIXED procedure). Generalized linear mixed models (ie, mixed-effects logistic models) were used to analyze dichotomous outcomes. Covariates included CKD stage at baseline, age, sex, smoking status, diabetes diagnosis, CKD diagnosis at baseline, ACEi and ARB at baseline, use of NSAIDs at baseline, blood pressure control at baseline, LDL control at baseline, HbA_1c_ at baseline (<7%, ≥7%, or test not done). Time since baseline (measured in days and converted to proportion of a year to aid interpretability) and intervention status were included as main effects, with differential change over time tested using a 2-way interaction effect (time × intervention). Model-based type 3 *F* tests for fixed effects were used to evaluate statistical significance of the intervention (ie, difference in slopes assessed time × intervention interaction). Additionally, we tested whether the intervention effect on change in eGFR differed for patients with CKD stage 3 vs stage 4 by adding a 3-way interaction term (time × intervention × stage) and all relevant 2-way interactions. Statistical significance was assessed using the model-based type 3 *F* test for the 3-way interaction term. For all tests, 2-sided *P* < .05 was considered statistically significant, and actual *P* values are reported in the tables. Analysis of SBP and HbA_1c_ over time used similar statistical modeling approaches. Analysis of dichotomous outcomes (use of NSAIDs, use of ACEi and ARB, CKD diagnosis during study period) used mixed-effects multilevel logistic regression (generalized linear mixed models); again, the model-based type 3 test for fixed effects was used to test for statistical significance.

The final sample of practices differed from the original set of randomized practices because of dropout. Therefore, we also performed analyses using propensity score methods for all outcomes. A propensity score for likelihood of receiving the intervention was computed for each patient and used as a covariate in the sensitivity analysis. Variables for the propensity score included CKD stage at baseline, age, sex, smoking status, diabetes diagnosis, CKD diagnosis, ACEi and ARB use at baseline, use of NSAIDs at baseline, blood pressure control at baseline, LDL control at baseline, and baseline eGFR. Secondary outcomes included SBP over time, HbA_1c_ over time, use of NSAIDs during the intervention period, use of ACEi and ARB during the intervention period, and CKD diagnosis recorded in the medical record (for patients without a CKD diagnosis at baseline). As described earlier, general linear mixed modeling was used for analysis of continuous outcomes. Generalized linear mixed modeling was used for dichotomous outcomes,^22,23^ adjusted for clustering of patients within practices. Analyses were performed using SAS version 9.4.^24^ Supplement 1 includes the data analysis plan.

## Results

**Figure 1.**
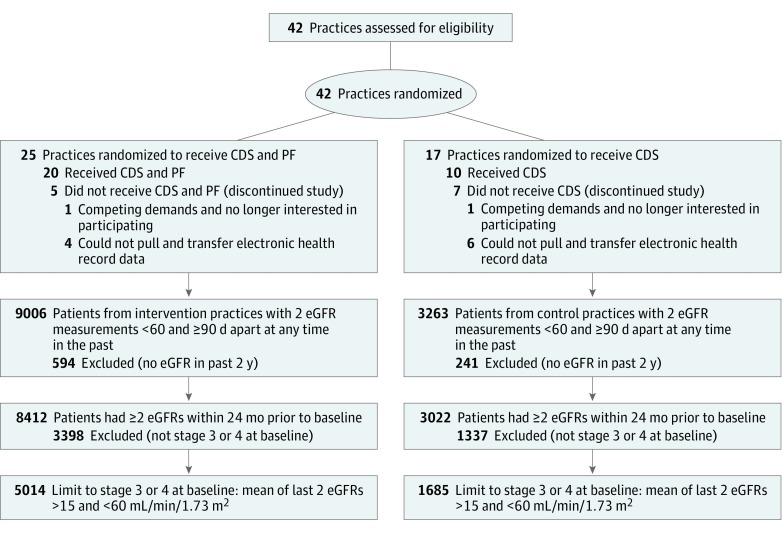
CONSORT Diagram Practices and patients from eligibility assessment through outcomes assessment. CDS indicates clinical decision support; eGFR, estimated glomerular filtration rate; and PF, practice facilitation.

Figure 1 shows the flow of participants (practices and their corresponding patient populations) through the study. Recruitment occurred from April 2012 to February 2014. The intervention was conducted from January 2013 to January 2016. Follow-up with sites was completed by January 2016. Patient data collection concluded in September 2015. The final cohort analyzed represented data from 30 practices (10 control and 20 intervention), with 1685 patients in the control group and 5014 patients in the intervention group (6699 patients total). Practices were located throughout the United States (see eFigure 2 in Supplement 2 for a map of practice locations).

Most of the practices that completed the study were family medicine specialty clinics (n = 22); the remainder were multispecialty (n = 7) and internal medicine (n = 1). Sixty-one percent of practices had 5 or fewer physicians. Most practices were physician-owned (90%). Patients in the practices were similar at baseline for age (mean [SD], 71.3 [9.6] years), sex (2716 male [40.5%]), and eGFR. Table 1 shows the baseline patient-level characteristics by intervention and control group practices. Patients in control and intervention practices that completed the study were similar for most baseline characteristics, including age, sex, baseline eGFR, blood pressure, HbA_1c_, creatinine, LDL, and current use of NSAIDs. As shown, control practices had patients with significantly greater frequency of diagnosis of CKD, frequency of diabetes, and use of ACEi and ARB medication at baseline compared with intervention practices. Practices that did not complete the study (n = 12) were similar to practices that completed the study for patients’ baseline clinical values (mean eGFR, *P* = .23; mean HbA_1c_, *P* = .46; and mean SBP, *P* = .48). There were no variables that could be assessed (ie, available for all 42 practices) that were associated with practice dropout.

**Table 1.** Baseline Characteristics of Study Cohort

Variable	No. (%)		
	Total Cohort (N = 6699)	Intervention Group (n = 5014)	Control Group (n = 1685)
Male	2716 (40.5)	1985 (40.0)	731 (43.4)
Age, mean (SD), y	71.3 (9.6)	71.4 (9.5)	70.9 (10.1)
Baseline eGFR, mean (SD), mL/min/1.73 m^2^^a^	47.1 (9.4)	47.9 (10.3)	46.3 (10.6)
Stage 4 CKD at baseline	2270 (33.9)	1626 (32.4)	644 (38.2)
Systolic blood pressure, mean (SD), mm Hg	130.7 (16.7)	130.8 (16.6)	130.5 (17.1)
Diastolic blood pressure, mean (SD), mm Hg	74.1 (10.1)	73.8 (10.1)	74.8 (10.3)
HbA_1c_, mean (SD), % of total hemoglobin^b^	6.81 (1.37)	6.79 (1.37)	6.87 (1.38)
Creatinine, mean (SD), mg/dL	1.35 (0.40)	1.33 (0.40)	1.40 (0.41)
LDL-C, mean (SD), mg/dL^c^	95.7 (34.4)	95.8 (33.9)	95.5 (35.8)
Current smoker	538 (8.0)	374 (7.5)	164 (9.7)
Diagnosed with CKD	1368 (20.4)	591 (11.8)	777 (46.1)
Diagnosed with diabetes	3160 (47.2)	2226 (44.4)	934 (55.4)
Receiving ACEi or ARB	3383 (50.1)	2167 (43.2)	1216 (72.2)
Current use of NSAIDs	1687 (25.2)	1236 (24.7)	451 (26.8)
Last blood pressure measurement ≤140/90 mm Hg	5144 (76.8)	3887 (77.5)	1257 (74.6)
Last LDL measurement <100 mg/dL	3777 (56.4)	2804 (55.9)	973 (57.7)
Last HbA_1c_ measurement			
<7.0% of total hemoglobin	2573 (38.4)	1879 (37.5)	694 (41.2)
≥7.0% of total hemoglobin	1258 (18.8)	888 (17.7)	370 (22.0)
Not done	2868 (42.8)	2247 (44.8)	621 (36.9)

Abbreviations: ACEi, angiotensin converting enzyme inhibitor; ARB, angiotensin receptor blocker; CKD, chronic kidney disease; eGFR, estimated glomerular filtration rate; HbA_1c_, hemoglobin A_1c_; LDL-C, low density lipoprotein cholesterol; NSAIDs, nonsteroidal anti-inflammatory drugs.

SI conversion factors: To convert HbA_1c_ to proportion of total hemoglobin, multiply by 0.01; LDL-C to millimoles per liter, multiply by 0.0259; eGFR to milliliters per second per meter squared, multiply by 0.0167; and creatinine to millimoles per liter, multiply by 88.4.

^a^ Mean of last 2 eGFR measurements prior to baseline.

^b^ n = 3831.

^c^ n = 6303.

Table 2 shows the results of the intent-to treat-and propensity-adjusted analysis of patients with CKD stage 3 and stage 4 from the final cohort of practices for the primary outcome: eGFR over time. There was no evidence to support a differential intervention effect for patients with CKD stage 3 vs stage 4 (*P* = .78), so this was not included in the final model. As shown in Table 2, there was a significant difference in eGFR slopes for patients in the intervention vs control group practices. In the control group, there was a mean (SE) decline of 0.94 (0.19) (*P* < .001) per year; there was no decline in eGFR for patients in the intervention group practices (slope [SE], −0.01 [0.02]; *P* = .91). Results from the sensitivity analysis using propensity scores were similar (intervention × time *P* < .001; slope [SE] in controls, −0.95 [0.19]; slope [SE] in intervention, −0.01 [0.12], mean [SE] difference in slopes, 0.93 [0.23]; *P* < .001). Thus, there was an annualized mean (SE) loss of eGFR of 0.95 (0.19) mL/min/1.73 m^2^ for control practice patients and a loss of 0.01 (0.12) mL/min/1.73 m^2^ for intervention practices.

**Table 2.** Intent-to-Treat and Propensity-Adjusted Analysis of Patients With Stage 3 and Stage 4 CKD From the Final Cohort of Practices on eGFR Change Over Time: Intervention vs Randomized Controls

Variable	Intent to Treat		Propensity Adjusted	
	Adjusted Models Coefficient (SE)	*P* Value	Adjusted Models Coefficient (SE)	*P* Value
Intercept	50.61 (0.51)		55.02 (0.73)	
Age	−0.21 (0.009)	<.001	−0.21 (0.01)	<.001
Sex				
Female	1 [Reference]		1 [Reference]	
Male	2.66 (0.17)	<.001	2.57 (0.16)	<.001
Current smoker	0.33 (0.30)	.27	0.20 (0.30)	.50
Diabetes	−0.49 (0.24)	.04	−0.65 (0.24)	.007
Receiving ACEi or ARB	−0.58 (0.17)	<.001	−1.46 (0.20)	<.001
Stage 4 CKD	−13.83 (0.20)	<.001	−13.76 (0.20)	<.001
CKD diagnosis	−2.64 (0.24)	<.001	−4.43 (0.31)	<.001
NSAIDs	0.96 (0.19)	<.001	0.82 (0.19)	<.001
BP ≤140/90 mm Hg	0.42 (0.19)	.03	0.50 (0.19)	.009
LDL-C <100 mg/dL	0.30 (0.17)	.07	0.38 (0.17)	.02
HbA_1c_				
<7% of total hemoglobin	1 [Reference]		1 [Reference]	
≥7% of total hemoglobin	−0.86 (0.24)	<.001	−0.76 (0.24)	.001
Not done	−0.66 (0.23)	.004	−0.76 (0.23)	.001
Intervention vs controls (at baseline)	−0.89 (0.50)	.08	−0.05 (0.56)	.93
eGFR mL/min/1.73 m^2^ change per 12 mo in controls (slope)	−0.94 (0.19)	<.001	−0.95 (0.19)	<.001
Difference in eGFR slope for intervention patients	0.93 (0.23)	<.001	0.93 (0.23)	<.001

Abbreviations: ACEi, angiotensin converting enzyme inhibitor; ARB, angiotensin receptor blocker; BP, blood pressure; CKD, chronic kidney disease; eGFR, estimated glomerular filtration rate; HbA1c, hemoglobin A1c; LDL-C, low density lipoprotein cholesterol; NSAIDs, nonsteroidal anti-inflammatory drugs.

SI conversion factors: To convert HbA_1c_ to proportion of total hemoglobin, multiply by 0.01; LDL-C to millimoles per liter, multiply by 0.0259; and eGFR to milliliters per second per meter squared, multiply by 0.0167.

Table 3 and eTables 3 and 4 in Supplement 2 show the results of the intent-to-treat and propensity-adjusted analyses of patients with stage 3 and stage 4 CKD from original randomized practices for the secondary outcomes. As shown in Table 3, there was a significant difference in HbA_1c_ trajectories, with greater decline in HbA_1c_ for patients in intervention practices compared with control group practices. Among patients with HbA_1c_ measures, slopes differed significantly for patients in intervention vs control practices, with a mean (SE) annualized increase of 0.14 (0.03) in HbA_1c_ for patients in control practices and a mean (SE) decline of 0.009 (0.02) for patients in intervention practices. There was a significant difference in HbA_1c_ slopes for patients in the intervention compared with control group practices (control vs intervention, −0.14; *P* < .001). There was no significant difference in slopes for patients in the intervention vs control group practices for SBP (eTable 3 in Supplement 2). As shown in eTable 4 in Supplement 2, there were no significant differences between the intervention and control groups for rates of avoidance of NSAIDs, use of ACEi and ARBs, and CKD identification (as noted by the presence of a CKD diagnosis on the problem list).

**Table 3.** Intent-to-Treat and Propensity-Adjusted Analyses of Patients With Stage 3 and Stage 4 CKD From Original Randomized Practices. HbA_1c_ Change Over Time: Intervention vs Randomized Controls

Variables for Patients With Stage 3 or 4 CKD With HbA_1c_ Values (n = 3850)	Intent to Treat		Propensity Adjusted	
	Adjusted Models Coefficient (SE)	*P* Value	Adjusted Models Coefficient (SE)	*P* Value
Intercept	5.82 (0.08)		5.98 (0.11)	
Age	−0.01 (.001)	<.001	−0.01 (0.001)	<.001
Sex				
Female	1 [Reference]		1 [Reference]	
Male	0.02 (0.03)	.48	0.01 (0.03)	.57
Current smoker	0.03 (0.05)	.48	0.03 (0.05)	.51
Diabetes	0.55 (0.03)	<.001	0.55 (0.03)	<.001
Receiving ACEi or ARB	0.06 (0.03)	.02	0.03 (0.03)	.30
Stage 4 CKD	0.05 (0.03)	.09	0.05 (0.03)	.07
CKD diagnosis at baseline	0.03 (.04)	.47	−0.04 (0.05)	.42
NSAIDs	−0.03 (0.03)	.37	−0.03 (0.03)	.29
LDL-C <100 mg/dL	−0.07 (0.03)	.007	−0.07 (0.03)	.01
Baseline HbA_1c_				
<7% of total hemoglobin	1 [Reference]		1 [Reference]	
≥7% of total hemoglobin	1.82 (0.03)	<.001	1.82 (0.03)	<.001
Intervention vs controls (at baseline)	0.04 (0.09)	.68	0.07 (0.09)	.45
HbA_1c_ change per year in controls (slope)	0.14 (0.03)	<.001	0.13 (0.03)	<.001
Difference in HbA_1c_ slope for intervention patients	−0.14 (0.03)	<.001	−0.14 (0.03)	<.001

Abbreviations: ACEi, angiotensin converting enzyme inhibitor; ARB, angiotensin receptor blocker; CKD, chronic kidney disease; HbA_1c_, hemoglobin A_1c_; LDL-C, low density lipoprotein cholesterol; NSAIDs, nonsteroidal anti-inflammatory drugs.

SI conversion factors: To convert HbA_1c_ to proportion of total hemoglobin, multiply by 0.01; LDL-C to millimoles per liter, multiply by 0.0259.

## Discussion

This study is significant in that it is one of the first large pragmatic clinical trials, to our knowledge, that took place in real-world primary care practices in which the outcomes for every patient with CKD within each practice were measured longitudinally. Because of its relatively new methodology, the study had important findings, limitations and strengths, and important lessons learned.

### Major Findings

**Figure 2.**
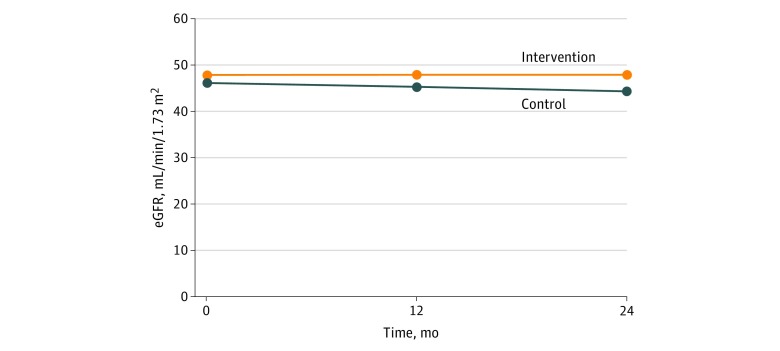
Model-Based Estimates of Estimated Glomerular Filtration Rate (eGFR) Over Time

In this multisite pragmatic cluster randomized clinical study, results showed a significant effect in the intervention group compared with the control group in slowing the rate of annualized loss of eGFR in patients with stages 3 and 4 CKD. The slower annualized eGFR decline in the intervention group (−0.01 mL/min/1.73 m^2^) vs the control group (−0.95 mL/min/1.73 m^2^) (or 0.94 mL/min/1.73 m^2^ difference) is associated with improved outcomes and cost reduction. A similar, although slightly more robust, finding was shown in the recently published EMPA-REG OUTCOME trial that demonstrated a significant adjusted mean difference between the drug-treatment groups vs placebo of 4.7 mL/min/1.73 m^2^ over a median 3.1 years of follow-up, or an annualized rate of 1.51 mL/min/1.73 m^2^.^25^ In addition, in the current study period (Figure 2), the mean eGFR CKD category progressed from CKD stage G3a (45-60 mL/min/1.73 m^2^) to CKD stage G3b (30-45 mL/min/1.73 m^2^) in the control population, while it remained the same in the intervention group. This preservation of kidney function is associated with lower all-cause and cardiovascular mortality,^26^ fewer cardiovascular events, lower 2- and 5-year subsequent risk of dialysis,^27^ and lower annual costs for Medicare ($10 088 vs $15 319) and commercial insurance ($14 263 vs $28 716) compared with declining kidney function.^28^ This eGFR finding was also observed among patients with diabetes or HbA_1c_ less than 7% of total hemoglobin at baseline. This result was observed despite controlling for imbalance in some baseline characteristics between the intervention and control groups and differential dropout of control practices. Additionally, there was significant improvement in HbA_1c_ over time among patients in the intervention group compared with the control group. There was no difference between groups in the remainder of the secondary outcomes—change in blood pressure, avoidance of NSAIDs, use of ACEi and ARBs, and rates of CKD identification (as noted by presence of a CKD diagnosis on the problem list).

The results of these findings imply but do not prove that control of diabetes is one of the more significant factors, if not the most significant, in helping to delay the progression of CKD. This is consistent with the findings that some of the new classes of drugs like sulfated glycoprotein-2 products delay the progression of CKD. Because of the imbalance that occurred in the dropouts in the control practices, the validity of these findings is a major question.

### Limitations

A limitation of this study is that many more control practices dropped out than intervention practices. One of the constrained variables used in initial randomization was baseline quality metrics, but when a number of control practices dropped out, the baseline quality of the control practices was superior to the intervention practices—calling into question the validity of the study’s results. We attempted to compensate for this by using propensity scoring. Whether one believes the conclusion in this study is valid will be based on their trust of the validity of propensity scoring to balance unbalanced groups.

We originally were going to use a single computer decision support system, but the vendor for the planned support system experienced economic difficulties. As a result, we had to use 3 separate systems, all of which were able to create registries and were able to create point-of-care computer decision support.

There were 2 major reasons that practices dropped out of the study. The first was that the practice or the organization that owned the practice changed its EMR system and the new EMR was not capable of even creating patient registries so that we could get the data to study our outcomes. We therefore had to drop them from the study. The second reason was that many small practices that had agreed to the study were bought by larger hospital groups and health care systems. The new owners of the practices then refused to let the practices continue in the study. In essence, we lost the *A* of TRANSLATE, administrative buy-in. We found administrative buy-in was a necessary factor in being able to retain practices. Learning to compensate for the potential ownership change was a major lesson we learned.

We can only speculate as to why the study became imbalanced after the practices that were bought by hospitals dropped out of the study. The buying up of smaller practices was clearly a secular trend that we had not anticipated when we began the study in 2011. Speculating on the reason, it seems plausible that if a hospital is buying practices within its community, the practices that are willing to sell out to the hospital might be the weaker practices and not the stronger ones. This could account for why we observed an imbalance in the remaining control group practices appearing better for some of the baseline CKD quality metrics.

### Strengths

This was a national multisite study with 30 practices and 7 different EMRs from which we were able to collect and aggregate data on more than 9000 patients. This was done without disrupting patient flow. Many of these patients who are cared for in small practices and in primary care offices are rarely studied as part of large randomized clinical trials. Additionally, there is a cost efficiency in doing this kind of study. This was a 5-year study that was funded for $2.5 million. By comparison, major randomized clinical trials such as ALLHAT (Antihypertensive and Lipid-Lowering Treatment to Prevent Heart Attack Trial)^29^ and CRIC (Chronic Renal Insufficiency Cohort Study)^30^ cost more than $100 million.

### Lessons Learned

We learned that it was feasible to do a large-scale study that was geographically widespread with primary care practices using multiple EMRs. We learned that we were able to collect large amounts of longitudinal data on a large number of patients in a Health Insurance Portability and Accountability Act–waivered manner that did not require the patient to either opt in or opt out. This was because the practice was the subject of the study. All patient measures were deidentified as a limited data set prior to leaving the practice’s EMR.

The second lesson learned was that randomizing at the practice level did not work if the practices were in the same organization that used a common quality improvement methodology. We had to adjust our randomization to block randomize at the organizational level. The need to balance practices and randomize practices in the real world became another problem that we addressed with the concept of constrained covariate analysis.^20^

A third lesson was that, given our inability to control for secular trends, a third study group would have been helpful. In this study, the secular trend of the buying up of small practices posed a major challenge. A third group, ie, a contemporaneous control group, where we measured practice data without intervening in the practice, would have greatly strengthened the study.

The problem of new owners buying practices and then mandating that practices drop out of the study was addressed by investigators in another study who learned from our study’s challenge. In that other study, the investigators included language in the contract that if the practice dropped out of the study because of change of ownership, the new owners will refund any grant monies they have previously received. It is hoped this will provide an incentive to continue in the study rather than withdrawing support for research at the practice level. Therefore, the study reported here paved the way for other pragmatic clinical trials in primary care offices. Its major value was in the lessons learned and the demonstration of feasibility.

## Conclusions

A multimodal intervention in primary care based on the TRANSLATE model slowed annualized loss of eGFR. This study had several important strengths, weaknesses, and lessons learned regarding the implementation of pragmatic interventions in primary care to improve CKD outcomes. This study is one of the first large pragmatic clinical trials we are aware of that took place in real-world primary care practices where the outcomes for every patient with CKD within each practice were measured longitudinally. This was a national multisite study, with 30 practices and 7 different EMRs from which we were able to collect and aggregate data on more than 9000 patients. A major problem in this study is that many more control practices dropped out than intervention practices. We attempted to compensate for this by using propensity scoring. Whether one believes the conclusion in this study is valid will be based on their trust of the validity of propensity scoring to balance unbalanced groups. Lessons learned from this study can inform other pragmatic clinical trials in primary care offices.

## Notes

Supplement 1. **Trial Protocol**

Supplement 2. **eTable 1.** TRANSLATE Elements as Implemented **eReferences** **eTable 2. **List of EHRs **eTable 3.** Intent to Treat Analysis Of Stage 3 and Stage 4 Patients From the Final Cohort Of Practices. Systolic BP Change Over Time: Intervention vs Randomized Controls **eTable 4.** Other Secondary Outcomes: Intervention vs Randomized Controls **eFigure 1.** Data Sources for the Creation of the Point of Care Reminder **eFigure 2.** Map of Practice Locations

Supplement 3. **Data Sharing Statement**
