# Supplementary material for: Effect of 2 Clinical Decision Support Strategies on Chronic Kidney Disease Outcomes in Primary Care: A Cluster Randomized Trial
Source: JAMA Netw Open. 2018 Oct 26;1(6):e183377. doi: 10.1001/jamanetworkopen.2018.3377 (PMC6324427; doi:10.1001/jamanetworkopen.2018.3377)
Supplement: Supplement 1. — Trial Protocol [file jamanetwopen-1-e183377-s001.pdf]

DATA ANALYSIS

General Approaches

Initially, descriptive statistics (mean, SD, proportions) will be computed for baseline patient and practice characteristics. In addition, chi-squares and t-tests will be used to determine whether there are differences between: (1) patients in practices randomized to different intervention conditions, and (2) dropouts and non-dropouts. Practices randomized to the two intervention groups (TRANSLATE, CDS only) will be compared on patient sociodemographic and clinical variables; these variables will be included as covariates in subsequent analyses if they differ between groups, are associated with outcomes, or are associated with dropout. In general, we will employ methods that utilize all available data, assuming ignorable missingness (MCAR or MAR).[54-59] For primary outcome variables that are continuous (or ordinal) we will explore whether these outcome variables are normally distributed prior to analysis. In the event that normality assumptions are not met, we will use transformations to normalize distributions, ordinal or Poisson regression where appropriate, or techniques using the appropriate link function (e.g. logit link for dichotomized measures).[60] We will employ intent to treat analyses using general (generalized) linear mixed model approaches (GLMMs) to incorporate data structures that are both hierarchical and longitudinal.[61] For time to event outcomes (e.g. death, ESRD), Cox proportional hazards models will be used to analyze the data. All hypothesis tests will be two-sided with alpha=.05 or p values reported). Goodness of fit statistics (e.g. AIC, deviance, -2 log likelihood and change in –2LL for nested models) and model fitting diagnostics to assess for influential points, outliers, overdispersion and heteroscedasticity will be used to evaluate alternative model specifications.[60] Covariates will be screened initially in bivariate analyses and included in multivariate analysis if they are related to the outcome at p<.2, differ between treatment arms, or are associated with dropout.

Because all data will be gathered from the practice EHRs, availability of data will not be dependent on participation in interventions, allowing robust estimates of effectiveness of interventions among those for whom they are intended as well as sub-analyses among those who participate. All statistical analyses will be performed using SAS version 9.2 (SAS Institute Inc., Cary, N.C.).

Specific Aim 1: Conduct a group randomized controlled trial of point-of-care computer decision support plus the full TRANSLATE mode of practice change, versus computer decision support alone in promoting evidence-based care in primary care practices for all patients with an eGFR <60 and > 15 ml/min/1.73m^2 (stage 3 and 4) confirmed with repeat testing over three or more months.

Power and Sample Size

With 20 practices per arm and a minimum of 200 patients per practice there will be a minimum of 4000 patients per arm. A sample size of 4000 per arm will provide >80% power to detect a .17 effect size difference between two arms at a single time point if the ICC is 3%. This effect size was assumed based on previous results from the diabetes TRANSLATE study.[2]In terms of change over time, a sample size of 4000 will provide >80% power to detect a small linear trend effect (increasing from 0 at baseline to .2SD at final follow up) with four observations per person and an ICC of 3%, with a random effects structure with random intercept and random slope and 5% attrition over time.[62] If the ICC is higher (e.g. 10%) and attrition is higher (e.g. 20%) we will still have power to detect a medium linear trend effect (increasing from 0 at baseline up to .5SD difference at final follow up) with four observations per person.[62]

Analysis plan for the TRANSLATE CKD Trial. Property of the authors. All rights reserved.

Patient Cohort for SA1 and SA2. Patients will be identified as eligible for this cohort if they meet criteria for stage three CKD at baseline; new patients will be added to the cohort up to *24 months* after initiation of the group-randomized trial to allow for potential minimum follow-up of 12 months. In the analyses described below, time will be coded individually for each patient, depending on when the patient is eligible to become part of the cohort. Diagnosis of stage 3 CKD requires two eGFRs<60 ml/min/1.73m^2 at least three months apart. For patients in the initial cohort time 0 is defined as the date of randomization; for patients added to the cohort time 0 is defined at date of the second eGFR<60. Therefore, baseline (time 0) will be defined as the date of randomization for patients who meet criteria for stage 3 CKD prior to study initiation or the *second* eGFR<60 with another eGFR <60 occurring a minimum of 3 months prior and no intervening eGFR>60 for patients who meet criteria for stage three CKD from baseline to 24 months after baseline. The rationale for choosing the latter definition for baseline for patients entering the cohort is based on the initial date when the physician would be expected to confirm the presence of stage 3 CKD and begin active clinical management to delay progression.

Hypothesis 1.1: CDS practices using the TRANSLATE model will provide a greater degree of evidence-based guideline-concordant care for CKD than CDS only practices.

The primary outcome for this analysis will be a patient-level score based on the percentage of goals achieved. Each goal will be assessed using EHR data for the *previous year (or part of the year in which the patient is eligible)* at baseline, 12 months, 24 months, and 36 months, as described in the table below. A composite guideline concordance score (GCS) will be created as the proportion of the number of applicable goals met. Secondary analyses will examine each outcome individually using all available data and continuous measures (e.g. systolic BP, HbA1c, LDL) or dichotomous measures (ACE/ARB, referral, smoking, NSAIDS).

TABLE 1: Evidence-Based Outcome Measures

Treatment Recommendation	Goal	Measurement
Control blood pressure	130/80	Means of last three systolic and diastolic BP; will be based on last one or two if fewer than three available
Control HbA1C	<7.0	Last HbA1c;
Control LDL	<100	Mean of last two LDL; last LDL if only one is available
Use ACE/ARB		Documentation in EHR/pharmacy of prescription; yes/no for each time period
Refer to Nephrologist (GFR < 30)		Referral documented, if applicable
Eliminate smoking		Yes/no for each time period
Eliminate NSAID/Cox-2 use		Yes/no for each time period

The structure of the data is hierarchical (patients nested within practices) and longitudinal (repeated assessments on patients at baseline, 12, 24, and 36 months).

Level 1 model. Repeated measures within each patient will be modeled as a time trend (linear growth curve shown below; quadratic trend will be tested) model. Time will be coded as days since baseline,

converted to months to aid interpretability. The guideline concordance score for patient i measured at time t in practice j is Y_{tij}

All rights reserved.

$$Y_{tij} = \pi_{0ij} + \pi_{1ij}\,(\text{time})_{tij} + \varepsilon_{tij}$$

where π_{0ij} is the individual status at time 0, π_{1ij} is the linear growth rate for patient ij, and ε_{tij} is the term that represents the random deviation of observation t within patient ij from the predicted value.

Level 2 model. The patient level models specify the relationship between the patient-level coefficients and the coefficients in the Level 1 model. Fixed patient-level clinical and sociodemographic covariates (X_j) will be included at this level.

$$\pi_{tij} = \beta_{t0j} + \Sigma\beta_{tpj}\,(X_i) + r_{tij}$$

where β_{00j} represents the initial status of patient i within practice j, β_{10j} represents the linear growth rate for patient ij and r_{tij} is a patient-level random effect.

Level 3 model. The practice level models specify the relationship between the practice-level predictors and the coefficients in the Level 2 model. TRANSLATE will be coded 1 for facilitated practices and 0 for CDS only practices.

$$\beta_{00j} = \gamma_{000} + \gamma_{010}\,(\text{TRANSLATE}) + u_{00j}$$

$$\beta_{10j} = \gamma_{100} + \gamma_{110}\,(\text{TRANSLATE})$$

where γ000 is the intercept in the practice level model for β00j (i.e. mean initial status for usual care practices, adjusted for individual level covariates); γ010 represents the mean difference at baseline between usual care and facilitated practices; γ100 is the linear growth rate for usual care practices, γ110 is the *difference* in linear growth rate for usual care vs facilitated practices.The u's are practice-specific random effects that represent the deviation of practice j's coefficient from its predicted value and are independent of r_{tij} and assumed to have a bivariate normal distribution over practices. Thus, the primary hypotheses of intervention effectiveness on guideline concordance can be tested as H_0: $\gamma_{110}=0$. Other hypotheses of interest can be tested using a priori specified linear contrasts.

Specific Aim 2: Conduct an intent-to-treat analysis between the CDS practices with facilitation versus the CDS only practices of the clinical outcomes of CDK progression and all-cause mortality.

Hypothesis 2.1: Patients with stage 3 and 4 CKD in facilitated practices will have slower CKD progression than patients in CDS only practices.

The outcome for this analysis will be eGFR measurements over time. There will be multiple eGFR measures per patient over the duration of the study. We will use general linear mixed-effects models to estimate the rate of decline in eGFR and the degree to which the baseline covariates predict eGFR. Time for each observation will be coded as days since baseline, converted to months to aid interpretability. The statistical model will be the same as described above for hypothesis 1.1. The primary hypothesis of difference in slope between treatment groups will be tested as H_0: $\gamma_{110}=0$.

Hypothesis 2.2: Patients with stage 3 and 4 CKD in facilitated practices will have significantly lower all-cause mortality than stage 3 and 4 patients in CDS only practices.

All-cause mortality will be confirmed using the National Death Index to determine the exact date of death. The outcome for the analysis will be time from baseline to death. Patients who are alive at the end of the study period will be censored at the end of the follow-up time. Assumptions of the proportional hazards model will be checked for each variable. Covariates will include baseline eGFR, defined as the mean of the last two eGFRs prior to study entry, as well as sociodemographic and clinical characteristics. The Cox models will be adjusted for

Analysis plan for the TRANSLATE CKD Trial. Property of the authors. All rights reserved.

clustering of patients within practice. To assess discrimination, we will calculate the *c*-statistic from the Cox regression models using methods described previously.[63,64] The *c*-statistic is equivalent to the probability that the predicted risk is higher for a case than a non-case and has a maximum value of 1.

 All rights reserved.

Institutional Review Board

Application for Review of Research Involving Human Subjects

General Information – Administrative Data	1.0
Note: Investigators and all other key personnel involved in this project must complete the Collaborative Institutional Training Initiative (CITI) Computer Based Training Course through the University of Miami. https://www.citiprogram.org/	

Application Type (select one)

____	New Study
____	Request for Exemption, please indicate category: ____
____	Amendment to Existing Approved Protocol. If requesting an amendment, please make changes to the currently approved IRB protocol using track changes. Please submit the currently approved protocol with an updated signature under the Investigator's Assurance, at the end of this form, to the IRB.
X	Continuation. Please use your currently approved protocol and complete **Appendix A** found at the end of the application. Do not submit Appendix A only.

Protocol Title

Please enter the title of your protocol. If you are submitting multiple grants (funding sources) for this protocol please choose a generic title that represents all of the funding sources. Each individual grant will be listed in the approval letter.

Improving Evidence-Based Primary Care for Chronic Kidney Disease

Duration of Study

Expected Project Start Date	March 1, 2012 for practice recruitment
Expected Project End Date	June 30, 2017

Principal Investigator

Name	Jennifer K. Carroll
Title/Appointment	AAFFP NRN Director
Degree	MD, MPH
Office Address	
School/Department/Organization	
Primary Phone	
E-mail Address	

Co-Investigator

**Name**	Kim S. Kimminau
Title/Appointment	Research Director of the AAFP NRN
Degree	PhD
Office Address	
School/Department/Organization	
Primary Phone	
E-mail Address	

Co-Investigator

Name	Miriam Dickinson
Title/Appointment	Biostatistician
Degree	PhD
Office Address	
School/Department/Organization	
Primary Phone	
E-mail Address	

Other Study Staff

Name	Brian Manning
Title/Appointment	Associate Research Director
Degree	MPH, CHES
Office Address	
School/Department/Organization	
Primary Phone	
E-mail Address	

Other Study Staff

Name	
Title/Appointment	
Degree	
Office Address	
School/Department/Organization	
Primary Phone	
E-mail Address	

12

Other Study Staff

Name	
Title/Appointment	
Degree	
Office Address	
School/Department/Organization	
Primary Phone	
E-mail Address	

13

Other Study Staff

Name	Gerald Pulver
Title/Appointment	Quantitative Analyst
Degree	PhD
Office Address	
School/Department/Organization	
Primary Phone	
E-mail Address	

14

Funding 2.0

15

Is this project funded?

_____ Yes X No

Name of Funding Source	National Institute of Diabetes and Digestive and Kidney Diseases (NIDDK)
Title of Grant	Improving Evidence-based Primary Care for Chronic Kidney Disease
Type of Funding (Internal or external)	External

16
17

Study Information 3.0

18

Primary Study Location

Name of Location: American Academy of Family Physicians National Research Network

Briefly describe what will happen at this site:

The AAFP NRN will participate/conduct the following activities:

- Practice recruitment, enrollment and ongoing engagement (including project-related communications, paperwork, payments, and so forth);
- Maintaining IRB compliance with the AAFP IRB;
- Practice facilitation (e.g., working with the intervention practices to implement CKD guidelines);
- Data mapping and extraction process with the clinical decision support vendor;
- Data management for the quantitative and qualitative analyses;

- Analyses, preparation, and dissemination of results.

19

Is this a multi-center project? A multi-center study is one where different PIs at different institutions are conducting the same study or aspects of the same study

X	Yes If yes, complete the following questions		No

20

List all Collaborating and performance sites

Name of Location(s)	University of Buffalo, Buffalo, NY is a collaborating site. Forty-two performance sites were enrolled. Sites are listed in Appendix B.
Select all that apply:	
X (University of Buffalo)	IRB approval provided by site. If yes, please provide a copy of the IRB approval letter/e-mail **– See Appendix C.**
	Letter of cooperation or support (as appropriate)
X (Performance sites)	IRB Agreement (site is relying upon this IRB approval)

What will happen at this site(s)?

Collaborating site: The study team based at the University of Buffalo will oversee the academic mentoring component of the intervention. This site will also participate in the practice facilitation, some data management for the qualitative data and process evaluation data, and the analyses, preparation and dissemination of results.

Performance Sites: As part of the enrollment process, the organization of each performance site will sign a Limited Data Use Agreement with the National Research Network/DARTNet, thus allowing the clinical decision support (CDS) vendor, CINA (or Other), to do the following:

- Extract a limited data set from each participating practice's clinical data repository,
- Aggregate the standardized data into a single database,
- Share with IRB-covered research team members.

Individuals in each performance site will also be asked to complete informed consents that allow for the practice personnel to participate in the process evaluation, including interviews, and practice surveys.

Once enrolled, the scope of activities conducted by each performance site on behalf of the study will depend on the random assignment. Performance sites in the CDS plus Facilitation arm will engage with study facilitators and academic mentors in order to implement the CKD guidelines proposed by the study protocol and supported by the CKD algorithms, feedback, and point-of-care reports provided by the CDS vendor (CINA or Other). The performance sites in the CDS-only arm will be encourage to implement the CKD guidelines and have identical CDS vendor support but they will not participate in the facilitation or mentoring process. However, they will still be asked to participate in the listed activities for the process evaluation, including the interviews, and practice surveys. The performance sites will rely on the AAFP IRB approval for coverage (through the individual investigator agreement) unless the site has its own IRB.

21

In carrying out this research project will you be collecting, reviewing or receiving "Protected Health Information"? Protected Health Information is individually identifiable health information transmitted or maintained in any form or medium, which is held by a "Covered Entity" or its business associate. A Covered Entity is a health plan, a health care clearinghouse, or a health care provider who transmits any health information in electronic form in connection with a HIPAA transaction, such as billing.

X	Yes If yes please provide as an attachment, information about the covered entity's policies and procedures regarding HIPAA compliance.		No

Each organization has its own HIPAA compliance guidelines. Within these guidelines moving data as either fully de-identified data or using a limited data use agreement are approved methods for data sharing. For this project the only PHI that will be moved is dates of service. This will be done under a limited data use agreement that follows the guidelines for all organizations as long as the data use agreement has been appropriately reviewed by an IRB. No other PHI will be moved. By signing the DUA, the organization gives the research team permission to view these data and use for research analysis. **Please see the Data Use Agreement in the Appendices** (previously submitted to IRB).

22

23

Summary of Proposed Research 4.0

24

Project Summary Provide a brief summary of the scope of work for this project, using non-technical terms that would be

understood by a non-scientific reader. This summary should be no more than 200 words.

This application represents the AAFP NRN's component of a larger NIDDK (NIH) grant awarded to the University of Buffalo in which the AAFP NRN is a subcontractor. The personnel at the AAFP NRN and the personnel at the University of Buffalo will work together in an integrated fashion; however, this IRB application only covers the activities and actions of the AAFP NRN research team. The personnel from the University of Colorado Denver and University of Kansas Medical Center are considered NRN personnel through existing service contracts with the AAFP NRN. The performance sites (i.e., the participating practices) for this study will be covered by the AAFP IRB, unless they are already covered under their own IRB.

The prevalence of Chronic Kidney Disease (CKD) is steadily increasing in the United States, causing significant morbidities and mortality. There is reasonable evidence that specific actions/guidelines implemented by primary care physicians may help delay CKD progression and reduce mortality. The availability of Clinical Decision Support (CDS) for CKD may help promote effective, evidence-based care. The present study will test the extent to which CDS plus practice facilitation promotes evidence-based care and improves the clinical outcomes of CKD progression compared to using a CDS system alone. This study will endeavor to enroll up to 44 practices associated with the Distributed Ambulatory Research in Therapeutics Network (DARTNet), a federated network of organizations that use Electronic Health Records (EHR); or, practices associated with CINA, the CDS vendor for this study. Data from patients with CKD stages 3 and 4 will be included in the limited data set. The key endpoints are death, need for renal dialysis, or significant progression of CKD. Intermediary outcomes include process measures such as medication compliance and smoking cessation. The practice facilitation intervention is based on a well-studied approach for implementing Wagner's Chronic Care Model, using the acronym of TRANSLATE (**T**eam approach, **R**eminder systems, **A**udit & feedback, **N**etworked information systems & registries, **S**ite coordinator (logistical/operational), **L**ocal clinician champion (academic), **A**dministrative oversight, support & resources, **T**arget, **E**ducation & evidence.) In a previous studies using facilitation, significant lasting improvements were observed in the clinical outcomes of patients with diabetes. We are interested if similar results will be seen using facilitation plus CDS systems in patients with CKD.

Purpose and/or Rationale for Proposed Research Briefly describe the purpose and background rationale for the proposed project as well as the hypotheses/research questions to be examined. Should be no more than 200 words.

Specific Aim 1: Conduct a group randomized comparator trial of computer decision support (CDS) plus the full TRANSLATE practice facilitation model, versus CDS alone in promoting evidence-based care in primary care practices for all patients with an eGFR <60 and > 15 ml/min/1.73m2 (CKD stages 3 and 4).**Hypothesis 1.1:** CDS practices using the TRANSLATE model will provide a greater degree of evidence-based care than CDS-only practices.

Specific Aim 2: Conduct an intent-to-treat and process analysis between the CDS practices with facilitation versus the CDS only practices of the clinical outcomes of CDK progression and mortality.

Hypothesis 2.1: Patients with stage 3 and 4 CKD in facilitated practices will have slower CKD progression than patients in CDS only practices.
Hypothesis 2.2: Patients with stage 3 and 4 CKD in facilitated practices will have significantly lower mortality than stage 3 and 4 patients in CDS only practices.

Hypothesis 2.3: The process evaluation will determine through qualitative methods the fidelity of the facilitated TRANSLATE program; find the challenges and enablers of the implementation process, the role of facilitation, and the contextual factors that contribute to TRANSLATE decisions and strategies; and translate lessons learned into pragmatic "best practices" for future facilitation and dissemination.

Methodology/Procedures Describe sequentially and in detail, all procedures in which the research participants will be involved, e.g., paper and pencil tasks, interviews, observations, surveys, questionnaires, reviewing private records/files, physical assessments, audiotaping and/or videotaping, time requirement including number of sessions, amount of time per session, and duration or period of time over which the research will take place, etc. For school-based research where

class time is used, describe in detail the activities planned for nonparticipants and explain where both participants and non-participants will be located during the research activities. Include a concise description of procedures, locations, time commitments, and alternate activities on the relevant consent and assent forms.

Practice-level data

Informed Consent:
Across both arms, all practices will need to present a Physician Champion and Study Coordinator who are willing to complete informed consents on behalf of the practices and complete the paperwork to serve as individual investigators for this project (unless covered by their own IRB). The project manager, research assistant, academic mentors, facilitators, and qualitative analysts will all interact with these two key practice members, and communications with these individuals will be considered part of the process evaluation data. NOTE: The comparator practices will not have contact with the facilitators and academic mentors. In later stages of the project involving interviews with other practice members, an abbreviated informed consent will be used. **Please see copies of the informed consents in the Appendices (previously submitted to IRB).**

Baseline Survey Assessment:
Across both arms (CDS-only and CDS plus Facilitation), members in each practice will be asked to complete a validated Clinician Staff Questionnaire short-form (CSQ), parts of the Office Vital Signs Survey (OVSS), and a brief survey regarding the practices' use of CINA (Other CDS). The burden time for this task is estimated at 5-10 minutes and completion of the surveys is completely voluntary on the part of each practice member. Survey respondents will be asked not to put any personal identifiers on the confidential surveys and each will be given an individual, self-addressed stamped envelope in which to return the surveys directly to the NRN offices. Consent is implied within the act of completing the survey. **Please see copies of the baseline surveys in the Appendices (previously submitted to IRB).**

Ongoing Assessment as part of Quality Improvement:
All practices will be asked to set CKD treatment targets and regularly review performance data to track progress. The intervention practices will be asked to facilitate practice innovations that improve the efficiency of CKD treatment and management; liaise with academic mentors and practice facilitators via videoconferencing and possibly face to face; and convene a regular quality-improvement team meeting to disseminate best-practice guidelines and share success strategies. Comparator practices will be given information (such as nationally-published guidelines) at baseline and encouraged to meet with practice teams as part of overall QI, using the same strategies as the intervention practices; however, they will not receive ongoing assistance. Telephonic and electronic communication between practice facilitators and the sites will be collected/documented to assess effectiveness of the facilitation program. To collect these data, practices will be notified in the informed consent and practice participation agreement that the facilitator will track all communication (phone, electronic, in person) and that this information will be considered confidential data by the research team. **Please see draft copies of the interviews in the Appendices (previously submitted to IRB).**

Patient-level data: The data set that is extracted by the CDS vendor (CINA or Other) and transferred to the research team will be a limited data set with no PHI except for dates of services associated with the variables described below. The limited data set extracted from practice EHRs will include the following, **as outlined by the Data Use Agreement (DUA) and DUA Amendment #1, which can be found in the Appendices.**

**Limited Data Set for Patient-Level Data**	
Year of Birth	Numerical
Gender	M/F
Race/Ethnicity	Standard major groups and Other
Current smoking	Current, never, past
Height and Weight/BMI	Hgt, wgt actual
Total visits/encounters	Encounter records
Hemoglobin	Numerical result
HDL	Numerical result
LDL-C	Numerical result
Triglycerides	Numerical result
Creatinine	Numerical result
AST	Numerical result
ALT	Numerical result

HbA1c	Numerical result
25 OH Vitamin D	Numerical result
Electrolytes	Numerical result
Serum Phosphorous	Numerical result
PTH intact	Numerical result
All meds	Coded (NDC)/RxNorm
All diagnosis – Active & Inactive	ICD-9
Blood pressure	Systolic and diastolic
Estimated GFR	Calculated value
Urine albumin/creatinine ration	Calculated value
Medicare Insurance Coverage	Flag for Medicare insurance
Nephrologists referrals	Referral records (when available)

Race and ethnicity may be available in the EHR as some practices collect this data, where these data are not available it will be imputed using validated algorithms from RAND Corporation.

32

Measures List all questionnaires, surveys, interviews, psychological measures, or other measures, that participants will be asked to complete. Submit labeled copies as an attachment to the application and indicate that the instrument is in the public domain or provide appropriate documentation of permission to use each scale.

The data to be obtained from the practice will include:

1. *Baseline survey set* – includes short-form Clinician Staff Questionnaire (validated survey in public domain), parts of the Office Vital Signs Survey (survey created by this research team currently undergoing validation process), and a short set of questions regarding the use of the clinical decision support system (questions developed by research team). All instruments can be found in the Appendices.
2. *Semi-structured or depth interviews* with physician champions, study coordinators, and possibly other key practice members at each practice; to be collected during ongoing phone interviews. Draft interview templates can be found in the appendices.
3. *Review of communication* between 1) practice and facilitator and 2) practice and academic mentor, and 3) practice and qualitative analyst (outside of schedule interviews). These communications may occur through the following methods: email, phone, conference calls, webinars,. Facilitators will be asked to log their communications with the practices. While it is possible communications between the project manager/assistant and practices are reviewed, it is less likely these communications will contribute to the process evaluation.
4. Review and analysis of minutes and notes from practice QI team meetings (either documented by practice member or facilitator).

The patient measures for the study will include:
The data set that is extracted by the CDS vendor (CINA or Other) and transferred to the research team will be a limited data set with no PHI except for dates of services associated with the variables described below. The limited data set extracted from practice EHRs is described in the Methodology/Procedures section.

33

Participants Involved in the Research Study **5.0**

34

Participation Population (select all that apply) If you select any on the list, please complete the Justification question below. If you select N/A from the list below, you do not need to complete the Justification question.

	Prisoners	X	Minors (Under Age 18) (Indicate Age Range)
	Institutionalized Residents	X	Physically or Mentally Challenged
X	Legally Incompetent	X	Employees or Subordinates of Investigators
X	Illiterate Participants	X	Public Officials or Candidates for Public Office
X	Employees/Agency Staff	X	Pregnant Women
	N/A (Participating Population is not identified as a vulnerable populations under the regulations)		

35

Justification

Please provide justification for using minors or vulnerable populations selected above.

This study is looking at a limited data set of all patients with a diagnosis of CKD. While unlikely, it is possible that the data set may incidentally include limited clinical data on patients who are part of these vulnerable populations. However, they are not being targeted for inclusion and we will not be able to identify any individuals whose data are included.

36

Number of Subjects

Please provide the anticipated number of subjects to be enrolled.	Practice-level data: In order for a practice to enroll, a lead physician and a site coordinator must sign an informed consent. With up to 44 practices, that is a minimum of **88 practice members** participating in the study evaluation. Participation by the rest of the practice members is voluntary. Each practice member will be asked to complete a baseline survey set, described in the Methodology section, of which completion will imply informed consent; and some members will be asked to participate in the interview process, of which brief informed consent will be obtained at the time. The exact number beyond the 88 original practice participants cannot be determined at this time since their participation will be voluntary.<br>Patient-level data: We anticipate that each of the participating practices will have average patient panels of approximately 5,500 patients with an average percentage of patients with Stage 3 or 4 CKD of 11 to 13%, resulting in approximately 605 to 715 eligible patients per practice who meet the inclusion criteria. This will result in a total patient sample of approximately 31,000 patients at baseline, with additional patients added over the course of the study. There are no limitations based on gender, race or ethnicity. At the inception of the study we will use data from the preceding 48 months to determine eligible patients with additional patients added to the analytical dataset as they meet study criteria after inception. All patient-level data are converted into a limited data set as part of the data extraction process.

37

Recruitment Process Specifically describe the step-by-step procedures for finding and recruiting research participants or requesting pre-existing data or materials. Name any specific agencies or institutions that will provide access. Identify who will contact prospective participants. Describe solicitation through the use of advertising posters, flyers, announcements, newspaper, radio television or internet, face-to-face interactions such as direct mail or phone contact, classrooms, subject pools, health care registries, and institutional "gatekeepers" as applicable. Attach a copy of any recruitment materials including: poster(s) advertisement(s) or letter(s) or solicitation scripts to be used for recruitment.

Recruitment of Study Patients: We will not recruit individual patients. The limited data set of the eligible patients in each practice will comprise the study patient panel.

Recruitment of Study Practices: For the trial we will recruit up to 44 practices already using CINA. Or Other CDS We will recruit practices in 3 stages:

- *Stage 1*: Email outreach for general interest only, followed up by postcard mailing to healthcare organizations already using CINA or Other CDS. This stage will help ascertain broad interest in the study and generate general awareness at the organizational level.
- *Stage 2:* Descriptive letter but no enrollment materials will be mailed to organizations that expressed an interest from stage 1. The goal of ascertaining early interest and generating awareness is to help organizations plan ahead in their quality improvement projects and annual QI plans, since the organization often plans the activities for the practices.
- *Stage 3:* Once IRB approval has been granted, formal practice recruitment will occur. An enrollment letter will be sent to practices that belong to organizations that expressed a previous interest. A webinar or phone conference will be arranged to review enrollment materials. Once all materials are reviewed and all questions answered, the practice will be allowed to formally enroll.
  NOTE: We anticipate great interest in this study and therefore more than 44 practices may express interest and wish to enroll. The practices selected for the study will be randomly selected from among eligible practices that agree to participate. The remaining practices will be put on the wait list. All payment issues will be fully explained to all practices during stage 3, including the fact that practices on the wait list will not be compensated unless they officially join the study at some point.

38

Will participants receive compensation for participation?

X Yes ______ No

If yes, please provide details including the form of remuneration including dollar amount, course credit, lottery, gift certificate. Explain the remuneration plan, including whether and how pro-ration will be made for partial participation. For lotteries include the number of prizes, nature and value of each prize. Include information about compensation on the

relevant consent and or assent forms. Please refer to “The Consent Process” guidance for more information.

This is a practice-level intervention; therefore the practices will be compensated as follows:
Comparator practices: payment of $250 per practice in Year 1, $750 per practice in each year for Years 2, 3, and 4 for a **total of $2,500 per practice** ($750 X 3 yrs = $2250 + $250 = $2500)
Intervention practices: payment of $500 per practice in Year 1, $1,000 per practice in each year for Years 2, 3, and 4 for a **total of $3,500 per practice** ($1000 X 3 years + $500 = $3500)
Wait-List practices: Practices that enroll but are not selected for randomization will be put on the waiting list. There is no compensation for being on the waiting list, but if selected for participation, the practice will receive the due compensation as the practice it is replacing, starting with the date of replacement.

Risk/Benefit 6.0

Potential Benefits from the Study Discuss any potential direct benefits to participants from their involvement in the project and/or the potential benefits to society that would justify involvement of participants in this study.

Benefits to Patients: Patients may experience a higher level of guideline concordant care for their CKD through the practice participation in this study.
Benefits to Practices and Practice Personnel: Practices and staff will better understand how they deliver care for patients with stage 3 or 4 CKD. Moreover, they also will learn how the potential enhancements in the care of these patients can be both attained and sustained over time. They will learn the potential barriers and facilitators for the delivery of quality care to patients with CKD and how to take advantage of the facilitators and ways to successfully address the barriers.
Benefits to Public Health: Completion of this research is expected to determine the value of facilitation in promoting evidence-based care for chronic kidney disease. It will also help determine the impact of greater guideline concordance on the rate of progression of CKD and explore the impact of several individual intermediate outcomes on the rate of progression of CKD. Ultimately, findings of the study will promote improved primary care for CKD.

Potential Risks from the Study Discuss the known and anticipated risks, if any, of the proposed research. Specify the particular risks(s) associated with each procedure or test. Consider both physical and psychological/emotional risks. Describe the procedures or safeguards in place to protect the physical and psychological health of the participants. e.g., referral to psychological counseling resources.

We do not foresee any major risks to patients as the goal of this study is to increase the use of evidence-based approaches to clinical care and all clinical decisions are in the control of the treating clinician and patient. Potential risk to patients is from loss of confidential data. Quantitative data will be aggregated at the entire data set level for all public presentations and the researchers will enter into limited data use agreements with each covered entity that contributes data to the study. This ensures no attempt will be made to identify or contact subjects. Risks to practice personnel may include:
Time and competing demands: The physician champion and/or site coordinator may experience some stress related to the competing demands of completing the research obligations and the normal course of clinical/administrative operations for patient care. Research project staff will be in contact with the practices on a periodic basis (typically weekly or bi-weekly) via phone and email; issues such as these (study burden, competing demands, and study responsibilities) will be addressed with the objective of decreasing any study-related burdens.
Knowledge of practice healthcare delivery: The site coordinator and physician champion - as well as other practice personnel -- may learn by their participation in the study that the quality of healthcare provided to their patients with stage 3 or 4 CKD does not meet the quality of care they previously thought they were delivering. This knowledge may result in disappointment, disbelief, discomfort, and/or feelings of failure. To the extent that a given practice’s reported data does not meet practice expectations, physician champions and study coordinators will be encouraged to discuss these issues with either Drs. Chet Fox or Joe Vassalotti, or the project facilitators as recommended by Drs. Fox and Vassotti.
Identifying practice clinicians & their healthcare delivery patterns:

Most data and provider reports will be aggregated at the practice-level and will not contain information on individual provider performance. However, in some cases, providers may request an individual provider report, as this information is helpful to providers in evaluating their own performance. This is a common request as a part of Quality Improvement activities within a practice. In the event such reports are requested, the PI will review the reports prior to distributing them to the providers. If there are any concerns over the information, the reports will be discussed privately with the providers in question. Any and all reports identifying practices by name and or individual provider will be solely for internal use among the study team and participating practices, for the purposes of the intervention. All external reports will be aggregated and anonymous and will not identify individual providers or practices by name. All providers asking for individual reports will sign a new consent that contains the information concerning risks and benefits of these reports.

43

**Consent**	**7.0**

44

Consent Process Describe when, where, from whom, by whom, and how often, voluntary informed consent will be obtained.

Informed Consent of Study Patients: No individual patient consent will be obtained since the intervention occurs at the practice level rather than at the patient level. All patient data will be viewed by the study team in a limited data set format.
Recruitment of Study Practices: For the trial we will recruit up to 44 practices using CINA or other CDS vendor. All recruitment will be by direct contact between study team personnel and decision-making personnel within each practice/organizations. Representatives from participating practices will be asked to sign two documents indicating consent:

- Practice Agreement: This "letter of agreement" outlines the expectations of the practice and its clinicians/staff and the expectations of the quality improvement team and the research team. While not a formal consent process and not a binding contract, this letter of agreement serves to clarify expectations for all parties involved. Practices will be randomly selected from among all eligible practices that sign this form.
- Informed Consent – Key Personnel: The Physician Champion and the Study Coordinator will be asked to complete an informed consent. Consent from both personnel is required to participate in the study.
- Informed Consent – Voluntary Personnel: All practice clinicians and staff will be asked to complete a confidential baseline survey (CSQ/OVS); completion is optional and implies consent. Throughout the study, study team staff and/or facilitators may conduct telephone interviews. At that time, practice staff that agrees to be interviewed will be asked to complete a brief Informed Consent or verbally give permission. Verbal permission will be audio-taped.

45

Waiver / Alteration of Informed Consent

Are you requesting a waiver or alteration of Informed Consent? X Yes ____ No

If you are requesting a waiver or alteration describe: (1) how the proposed research presents no more than minimal risk to participants, (2) why a waiver or alteration of informed consent will not adversely affect the rights and welfare of participants, (3) why it is impracticable to carry out the research without a waiver or alteration of informed consent. Also describe how pertinent information will be provided to participants, if appropriate, at a later date. Describe how you will otherwise fully inform participants, i.e., use of an information script, information letter, etc.

A waiver of informed consent is requested for patients, but not for clinicians / staff within the practice. Clinicians and staff will be consented, but it is not necessary or practical to consent patients whose limited data will be included in the aggregated data. Risk to patients is minimal as the research team is only able to obtain aggregated, limited data subject to the Limited Data Use Agreement. Data from health records is routinely extracted for quality improvement, financial and other purposes - and no consent is required. The data for this study will extend back four years from the start of the study, thus it is not possible to contact all the individuals who may be included in the aggregated data.

46

Research involving minors, or others who are not competent to give legally valid consent.

If you are involving minors, or others who are not competent to give legally valid consent, please explain how the subject's understanding will be assessed and how often. Include the questions that will be asked or actions that will be taken to assess understanding. Describe the process to be used to obtain permission of parent or guardian. Attach a copy of an information-permission letter to be used. **If you are not involving minors, or others who are not competent to give legally valid consent, you do not need to complete this question.**

Minors may be incidentally included in the limited data set, but we are requesting a waiver of informed consent.

47

Assent

if you are involving people who are not legally competent to give consent but are reasonably competent to decide whether to participate or not, describe the procedure you would use to gain assent and attach the form. Children must assent (or, voluntarily agree) to participate and a parent must separately provide permission on behalf of his/her child. Two separate forms are required. Children under age 7 may assent either orally or passively, depending on their level of maturity. **If you are not involving needing to assent anyone in your study, you do not need to complete this question.**

N/A

48

Will you be obtaining consent/assent from non-English speaking participants:

____ Yes X No

If yes, describe the process that will be used to translate documents, the language and qualifications of the translator.

49

Privacy and Confidentiality 8.0

50

Describe the procedures to be used to ensure the privacy of participation and data obtained.
Privacy is required unless subjects give express, written permission to have their identifiable information published, presented, or shared. Explain who will have access to raw data, whether raw data will be made available to anyone other than the Principal Investigator and immediate study personnel (e.g., school officials, medical personnel, federal agencies etc.) If yes, who, how and why? Describe the procedure for sharing data. Describe how the research participant will be informed that the data may be shared. Describe any circumstances under which you might be required to break confidentiality. Explain how you will inform potential subjects that confidentiality may be broken.

Collection of Data: All patient data will be collected from participating practices by CDS vendor (CINA or Other) as a limited data set or matched to the National Death Registry using standard operating procedures for DARTNet and CDS vendor. DARTNet is the electronic network associated with the AAFP NRN. Data from multiple sources within the EHR are initially stored in the CDS vendor Clinical Data Repository (CDR) for clinical care. Relevant study data are standardized within the CDS vendor CDR across all DARTNet sites and then aggregated into one large study database with dates of birth transformed to year of birth. All years of birth prior to 89 years ago will be converted to a single category.

Access to Data: Identified patient-level data will never be in the possession of the research team. After appropriate IRB approvals and data use agreements are obtained, the limited patient level data will be transferred to the investigators via secure FTP (File Transfer Protocol) from CDS vendor to a secure, DARTNet, research server maintained by the University of Colorado Denver for DARTNet studies. Research access to this server is closely monitored and limited to appropriate research team members.

Protection of Patient Data: Patient level limited data can only be extracted from each practice CDR on the positive action/approval of a designated individual within each practice. Direct identifiers never leave the practice. The limited data, once extracted, are transferred using secure FTP. Data forwarded to the research team will be housed on a secure research server at the University of Colorado Denver (UCD). UCD provides academic support to the AAFP NRN through a service contract that includes part time staff (including Drs. Pace, Dickinson and Pulver) as well as informatics support. Study data will be housed on research specific servers that are not only behind the UCD fire wall, but visible only to select IP (Internet Provider) groups within the overall UCD network. In addition, these data are stored on directories with limited user access. Data servers are managed by the UCD Department of Family Medicine, which includes a locked, environmentally controlled, 24-hour monitored server farm with redundant backup systems. Analysis will be performed on UCD SAS severs maintained by the campus information services, located on core or local workstations maintained by the Department of Family Medicine. No data will be stored on local workstations. The SAS servers are specifically maintained for secure analysis of health care data.

Protection of Practice-Level Data: *Because practice-level data is being used in a formative evaluation, it is necessary to retain the linkages between the practice and the data in order to provide information to the facilitators and academic mentors to guide the intervention. Practice-level data will only be shared internally within the research team.*

In all reports that are disseminated externally, there will be no link between the research data and identifiable participant information; all data will be completely de-identified with regard to provider and practice name. The study subjects in this proposal are the provider participants. All research team members will comply with federal regulations to ensure that confidentiality is maintained. There will be no reference by name of any provider participant (or their patients) in any external qualitative data report. All study records will be encoded with a unique participant number (UPN).

51

Data 9.0

52

Check if any of the following will be used in Data Collection:

X	Audio Tapes	X	Video Tapes
X	Still Photos		Other Imaging
	None	It is standard practice to audiotape interviews over the phone in order to preserve the fidelity of the interaction and also document the participant’s verbal informed consent. Interviews will only be recorded with permission of the interviewee. It is also typical to capture photos of the practice and/or personnel. Any personnel in photos will be asked to complete a photo release form in the case that a photo is later shared in a presentation, etc. Finally, since much of the facilitation efforts will be done virtually, it is possible that practices may send video of themselves doing a group exercise for the facilitator to review. Such videos could be considered part of the process evaluation.	

X Other (please explain)

53

Explain how the data will be kept confidential, stored, and disposed If anonymous data collection is proposed, provide details of how investigators will not have the ability to trace responses to research participants identities. For multiphase data collection or if multiple contacts will be made with research participants, specifically explain the tracking and coding systems that will be used. Address the confidentiality of data collected via e-mail, databases, Web interfaces, computer servers and other networked information, as applicable.

Patient-level data: There will be no contact between the research team and the patients whose limited data appears in the data set. When CDS vendor extracts and aggregates the patient level data, each patient record will be assigned a new, randomly generated GUID (Globally Unique Identifier) for this research project. Only members of the research team will have access to the organization and practice level codes. Only the local organization/practice and CDS vendor will have access to identifiable patient level data and this information will never leave the individual organizations/practices nor can any identified data be returned or even viewed by the research team even as it resides within the organization. The limited data set of research data will be stored on a secure server at the University of Colorado Denver. In publications and presentations, all data will be reported in aggregate and no individual will be identified.

Practice-level data: Practice-level data regarding clinical and personnel operations will be part of the overall qualitative and process evaluation. Depth interviews and semi-structured interviews from study coordinators, physician champions and other practice personnel will be collected during phone interviews. In addition, facilitators will document and retain a record of all communications with the practice, including phone, electronic, person, and through webinar or webcast. These data will be stored on a password-protected server, only available to the internal study group. The server is behind firewalls at UCD.

Qualitative data will be transmitted among study team members using secure email, a secure Survey Monkey account and / or upload and download via the secure server at UCD. Qualitative data comes from pre-existing emails and online web conferences between practice facilitators, academic mentors and practice staff, and therefore emailing this information among study team members does not increase the risk to study participants. Additionally, the information contained in this data is not sensitive or confidential in nature and consists mainly of communication regarding scheduling and organizing the facilitation, and general notes on high-level practice activities that do not identify individual providers' practice patterns. Practice personnel have consented to have their communications shared as part of the study data. No practice-level data will be identified outside of the internal study group unless explicit permission is granted by the practice in a voluntary effort; e.g., a high-performing practice might choose to share their "best practices" with others in the spirit of collaborative learning.

54

Deception **10.0**

55

Will participants be deceived or be incompletely informed regarding any aspect of this study?

____ Yes X No

If your response is "yes", describe the type of deception you will use, indicate why it is necessary for this study, and provide a copy of the debriefing script you will use with research participants explaining when and how it will be used.

56

Financial Disclosure **11.0**

57

Could the results of the study provide a potential financial gain to you, a member of your family, or any of the co-investigators that may give the appearance of a potential conflict of interest?

____ Yes X No

If yes and the financial interest will exceed $5,000 in a year, a financial disclosure statement is required with the application. Please contact the IRB for a copy of the financial disclosure form.

58

Investigator's Assurance **12.0**

I understand that as Principal Investigator, I have ultimate responsibility for the protection of the rights and welfare of human subjects and the ethical conduct of this research protocol. I agree to comply with all IRB and Institutional policies and procedures, as well as with all applicable federal, state, and local laws regarding the protection of human subjects in research, including but not limited to:

- Ensure that the project will be performed by qualified personnel according to the research protocol;
- Maintain a copy of all questionnaires, survey instruments, interview questions, data collection instruments, and information sheets for human subjects for at least three years following termination of the project except identifying links.

I have read and understand the above policy concerning IRB exempt protocols.

Signature of Principal Investigator	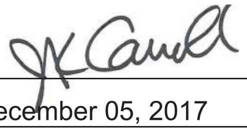
Date	December 05, 2017

Appendix A - Continuation

Complete Appendix A if you are requesting a continuation to a currently approved protocol. If you wish to amend the protocol as well, please make changes to the already approved protocol by using "Track Changes". Submit both the amended protocol and Appendix A to the IRB, via e-mail, at the same time. If there are no changes to the project, please submit the currently approved protocol with an updated signature under the Investigator Assurance and Appendix A, to the IRB, at the same time.)

Status of the project

	Study has not begun		Subjects still being recruited
	Subjects still being followed	X	Data analysis only
	Project Closeout: Data de-identified		Project Closeout: Link file destroyed

Clinician Participants*

Complete the chart below with the numbers participants *enrolled in the study to date:* (If this study collects racial, ethnic or gender identification please include it below. If not, complete "Totals" column only.)

Gender	Ethnicity						Total
	Asian	Black, not of Hispanic Origin	Hispanic or Latino	White, not of Hispanic Origin	Other	Unknown	
Male							
Female							
Unknown						50	50
Totals						50	50

Number of patients with baseline data

Complete the chart below with the numbers participants *enrolled in the study to date:* (If this study collects racial, ethnic or gender identification please include it below. If not, complete "Totals" column only.)

Racial Categories	Ethnic Categories									Total
	Not Hispanic or Latino			Hispanic or Latino			Unknown/Not Reported Ethnicity			
	Female	Male	Unknown/ Not Reported	Female	Male	Unknown/ Not Reported	Female	Male	Unknown/ Not Reported	
American Indian/Alaska Native	26	8	0	95	58	0	1	3	0	191
Asian	189	109	0	2	0	0	4	6	0	310
Native Hawaiian or Other Pacific Islander	6	6	0	0	1	0	1	0	0	14
Black or African American	1663	776	0	367	121	0	117	57	0	3101
White	8153	4120	0	267	133	0	490	261	0	13424
More than One Race	0	0	0	0	0	0	0	0	0	0
Unknown or Not Reported	93	60	0	38	18	0	3909	2467	0	6585
Total	10130	5079	0	769	331	0	4522	2794	0	23625

Have there been any unanticipated problems or adverse events involving risks to subject or others?

	Yes (please explain)	X	No

73

Have subjects withdrawn from the research?

X Yes (please explain, include number of withdrawn subjects) ____ No

There were no withdrawals since the last continuing review report.

74

Have there been any complaints about the research?

____ Yes (please provide a summary of any complaints) X No

75

Has there been additional or new information about this study which may affect the risk/benefit for subjects, which may affect a subject's willingness to continue participation to be given to prior participants?

____ Yes (please provide a summary) X No

76

Provide a brief report summarizing research performed in this study since the last IRB review, including progress of the research and preliminary information about results and/or trends?

All data collection and follow-up has concluded. Two presentations were added to the publications list (see page 16). We are currently working on the main outcomes manuscript. Analyses of two primary clinical outcomes (eGFR and systolic BP) are complete. The final patient cohort analyzed represented data from 30 practices (10 Clinical Decision Support and 20 Clinical Decision Support +Practice Facilitation). Primary measure: change in estimated glomerular filtration rate (eGFR) over time. 1) Significant difference in eGFR slopes. Control group: decline of ~0.83 per year; no decline for intervention group. Results from sensitivity analysis using propensity scores were similar. 2) No evidence supporting differential intervention effect for stage-3 vs. stage-4 patients (p=.7794) Secondary outcomes: Systolic blood pressure changes over time, nephrology referral for stage-4 CKD patients, avoidance of NSAIDS, use of ACE/ARBs, CKD diagnosis.

Comparing groups: 1) There were no significant difference in slopes for SBP change. 2) Fewer nephrology referrals, regardless of whether patient had baseline nephrology referral (31.8% vs 16%). 3) Nephrology referral likelihood significantly lower for patients not having referral at baseline (p<.0001). 4) No significant differences on rates of ACE/ARBs use, avoidance of NSAIDS, and CKD diagnosis Conclusions: Significant effect in slowing rate of progression of kidney disease in patients with stage-3 and stage-4 CKD observed despite controlling for imbalance in some baseline characteristics between the groups, and differential drop-out of control groups. No difference in secondary outcomes.

77

78 **Note:** When submitting this appendix with the updated protocol, please include the original copy of your consent form and
79 any proposed consent form changes as well as any investigator brochures describing the project.

Appendix B. Performance Sites (Last Updated 11-16-15)

Randomization Group	Organization name (w/ number of sites)	Group	Comments
**1st Randomization Pool**	**12 organizations (18 sites)**		
Randomized Fall 2012		C	
		T	
		C	Withdrew - Lack EHR capacity to participate
		T	
		T	
		T	
		T	Withdrew - Lack EHR capacity to participate
		T	
		C	
		T	
		C	Withdrew - Lack EHR capacity to participate
		C	
**2nd Randomization Pool**			
Randomized April 2013		C	
		T	1 Wilmington site withdrew because of competing demands
		C	
		C	Withdrew because of competing demands
		T	
**3rd Randomization Pool**			
Randomized March 2014			
		T	
		C	Withdrew - Lack EHR capacity to participate
		T	
		C	Withdrew – Lack EHR capacity to participate
**4th Randomization Pool**			
Randomized July 2014		C	
		T	
		T	
**TOTAL Enrolled**			
**TOTAL Dropped (after randomization)**			
**TOTAL Active**			

Appendix C. List of Publications and Presentations

2017

Carroll J, Pulver G, Dickinson M, Pace W, Vassallotti J, Knox L, Manning B, Bullard E, Smail C, Fox C. TRANSLATE CKD: A Cluster Randomized Trial Comparing Two Strategies to Improve Chronic Kidney Disease Outcomes in Primary Care. Oral presentation at NAPCRG Annual Meeting, November 17-21, 2017. Montreal, Quebec.

2016

Satchindanand N, Withiam-Leitch M, Dickinson M, Bublitz-Emsermann C, Allen GM, Yang M, Vassalotti J, Arora P, Glasgow P, Fox C. Positive Predictive Value of a Single Assessment of Estimated GFR in the Diagnosis of Chronic Kidney Disease. *Southern Medical Journal* 2016 June; 109(6): 351-5.

2015

Pulver G, Fox CH, Pace W, Brandt E, Dickinson LM. Putting together a functional longitudinal database structure from diverse electronic medical records for use in pragmatic clinical trials. Poster presentation at 8th Annual Conference on the Science of Dissemination and Implementation, Washington, D.C., December 14-15, 2015.

Fox CH. The promises and pitfalls of using clinical data from disparate electronic medical records for dissemination and implementation of evidence based chronic kidney disease care - Lessons learned from the translate CKD study. Poster presentation Poster presentation at 8th Annual Conference on the Science of Dissemination and Implementation, Washington, D.C., December 14-15, 2015.

Fox CH, Stuart B, Bullard E, and Turmiel-Berhalter L. "Engaging Patients in Research: Methods and Lessons Learned." Workshop presentation at North American Primary Care Research Group (NAPCRG), October 24-28, 2015. Cancun, Mexico.

Fox CH, Birtwhistle R, and Tobin J. "Working with National Federated Databases as a Means of Facilitating and Accelerating Research." Workshop presentation at North American Primary Care Research Group (NAPCRG), October 24-28, 2015. Cancun, Mexico.

Cipparone CW, Withiam-Leitch M, Singh R, Kimminau K, Fox CH, Kahn LS. Accuracy of ICD-9 Codes for Early-stage Chronic Kidney Disease: A Practice-based Research Network Study. *Journal of the American Board of Family Medicine* 2015 28 (5).

Dickinson LM, Beaty B, Fox CH, Pace W, Dickinson WP, Emsermann C, Kempe A. Pragmatic Cluster Randomized Trials Using Covariate Constrained Randomization: A Method for Practice-based Research Networks (PBRNs). *Journal of the American Board of Family Medicine* 2015 28 (5).

Kahn LS, Vest BM, Madurai N, Singh R, York TR, Cipparone CW, Reilly S, Malik KS, Fox CH. Chronic kidney disease (CKD) treatment burden among low-income primary care patients. *Chronic Illn* 2015 Sept. 11(3).

Vest BM, York TRM, Sand J, Fox CH, Kahn LS. Chronic Kidney Disease Guideline Implementation in Primary Care: A Qualitative Report from the TRANSLATE CKD Study. *Journal of the American Board of Family Medicine* 2015 28 (5).

Fox CH and Pace W. DARTNet Guided Exploration of Linkages Between Existing Health Data, Patient Reported Outcomes and PBRN Research. Webinar panel discussion for AHRQ PBRN Resource Center. August 7, 2015.

Neuhaus AK, Hall VM, Nguyen VT, Wisniewski AM. Virtual practice engagement in the TRANSLATE-CKD Study. Poster presentation at NAPCRG Practice-Based Research Network Conference, Bethesda, MD, June 29-30, 2015.

Nguyen VT, Neuhaus AK, Hall VM. Development of a Modified TRANSLATE Assessment Tool for the TRANSLATE-CKD Study. Oral presentation at NAPCRG Practice-Based Research Network Conference, Bethesda, MD, June 29-30, 2015.

Satchidanand N. The Positive Predictive Value of a Single Assessment of Estimated Glomerular Filtration Rate in the Prediction of Chronic Kidney Disease. Poster presentation at Translational Science Meeting, Washington, D.C., April 16-18.

Smail C. Comparing chronic kidney disease profiles identified using diagnostic histories and clustered clinical data with an established genetic risk model. Poster presentation at Biomedical Computation at Stanford 2015 Symposium, Stanford, CA, April 5-7, 2015.

2014

Kahn LS, Vest BM, Cipparone C, Madurai N, York TM, Malik KS, Fox CH. Addressing patient CKD self-management in underserved primary care settings. Oral Presentation. 42nd Annual Meeting of North American Primary Care Research Group (NAPCRG), November 21-25, 2014. New York, NY.

Neuhaus AK, Hall VM, Nguyen VT, Ramos NP, Vest BM, Cipparone C, Sand J, Talbot B, Bullard EM, Kahn LS. "Virtual Facilitation in the TRANSLATE-CKD Study." Poster presentation at North American Primary Care Research Group (NAPCRG), November 21-25, 2014, New York, NY.

Neuhaus AK, Nguyen VT, Hall VM, Ramos NP, Bullard EM. "Navigating Virtual Facilitation." Workshop presentation at North American Primary Care Research Group (NAPCRG), November 21-25, 2014. New York, NY.

Nguyen V, Neuhaus K, and Hall V. An Inside Look into the World of Virtual Practice Facilitation. Oral Presentation in the Best of Innovations in PBRN Methodology Category at the North American Primary Care Research Group (NAPCRG) Practice-Based Research Network Conference. June 30 – July 1, 2014. Bethesda, MD.

Vest BM, Talbot B, Kahn L, Cipparone C, Sand J, and Fox C. Evaluating Virtual Practice Facilitation and Practice Transformation in a Multi-site Trial to Improve Primary Care Providers' Recognition and Treatment of Chronic Kidney Disease (TRANSLATE CKD). Poster presented at the North American Primary Care Research Group (NAPCRG) Practice-Based Research Network Conference. June 30 – July 1, 2014. Bethesda, MD.

2013

Fox CH, Neuhaus K, Vassalotti JA. Importance of urine albumin–creatinine ratio in the diagnosis and prognosis of chronic kidney disease. *OA Nephrology* 2013 Nov 01;1(3):21.

Fox CH, Vest BM**,** Kahn LS, Dickinson LM, Fang H, Pace W, Kimminau K, Vassalotti J, Loskutova N, Peterson K. Improving Evidence-Based Primary Care for Chronic Kidney Disease: Study protocol for a cluster randomized control trial for translating evidence into practice (TRANSLATE CKD), (2013) *Implementation Science,* 8: 88. DOI: 10.1186/1748-5908-8-88.

Hunt S, Vest B, Neuhaus K, Hall V, Kimminau K, Fox C, Kahn L. "Process evaluation of a multisite translational research study to improve clinical outcomes in patients with Stage 3 and 4 chronic kidney disease (TRANSLATE CKD)." Poster

presentation. 41st Annual Meeting of North American Primary Care Research Group (NAPCRG). November 9-13, 2013, Ottawa, ON.

Kahn LS, Cipparone C, Snyder M, Withiam-Leitch M, Kimminau K, Duff B, Hake J, Zaudke J, Fox C. Misclassification of patients with chronic kidney disease (CKD): exploring diagnostic coding patterns in primary care. 41st Annual Meeting of North American Primary Care Research Group (NAPCRG). November 9-13, 2013. Ottawa, ON.

Kahn LS, Vest BM, Hunt S, Kimminau K, Neuhaus K, Fox CH. “Diagnosis and Treatment of Chronic Kidney Disease (CKD) in the Primary Care Setting: Physician’s Approaches and Challenges.” Poster Presentation. 41st Annual Meeting of North American Primary Care Research Group (NAPCRG). November 9-13, 2013, Ottawa, ON.

2012

Kahn LS, Vest BM, Sessamen S, York T, Stuart B, Tumiel-Berhalter L, Fox C. "Tell Me More:" Patients' knowledge of CKD and health information preferences. 40th Annual Meeting of North American Primary Care Research Group (NAPCRG). December 1-5, 2012, New Orleans, Louisiana.

Smail C, Loskutova N, Pace W, Fox CH, Stewart E, Bryan C. Is The Practice Recruitment Well Running Dry? A Case Study. 40th Annual Meeting of North American Primary Care Research Group (NAPCRG). December 1-5, 2012, New Orleans, Louisiana.

Wearner R, Hunt, S. Practices Learn the Most from Each Other: DARTNet Institute’s Approach to a Learning Community. Conference on Practice Improvement (CPI). November 29-December 2, 2012, Greenville, South Carolina.

York T, Kahn LS, Vest B, Fox C, Sessamen S, Snyder M, Abou-Jaoude. How CKD information should be delivered to me: insights on patient understanding and preferences for CKD diagnosis. 40th Annual Meeting of North American Primary Care Research Group (NAPCRG). December 1-5, 2012, New Orleans, Louisiana.

 IRB Application – TRANSLATE CKD Trial. Property of the Authors. All rights reserved.

REVISED TRANSLATE scoring tool 3-11-2015.xlsx

		TRANSLATE CKD rubric element	Score	Comments
		1 = No activity 2 = Activity, incomplete 3 = Complete 4 = Exceeds expectation (comment required)		
T	M0	Target measures	0	
	M1	Clinician/Team selects a sub-set of study performance measures		
	M2	Sets realistic goals for each selected measure		
	M3	Develops PDSAs to achieve each of the set goals		
	M4	Engages in iterative, performance-based target selection		
	M5	Integrates CKD measures with other ongoing initiatives		
R	R0	Reminders (clinical decision support)	0	
	R1	Practice has a functioning CDS		
	R2	Practice maps workflow and defines roles to use CDS		
	R3	Providers use CDS with all patients		
	R4	Monitors use of CDS by designated staff		
	R5	Develops capability to create POC alerts		
A	A0	Administrative Buy-in	0	
	A1	Perceived value by administration regarding study goals and objectives		
	A2	Commits resources necessary to accomplish study objectives		
	A3	Attends (or delegates) regular study team meetings		
	A4	Complies with agreed-upon study activities/tasks		
	A5	Maintains continuity of study personnel and tasks over time		
N	N0	Network Information Systems (CKD registry)	0	
	N1	Selects a registry system (paper, electronic, middleware product)		
	N2	Populates CKD registry		
	N3	Develops workflow to maintain and support registry function		
	N4	Uses CKD registry for population health management		
	N5	Monitors use of registry by designated staff (oversight)		
S	S0	Site coordinator	0	
	S1	Site coordinator identified		
	S2	Site coordinator meets regularly with facilitator		
	S3	Site coordinator works on project outside meetings		
	S4	Site coordinator facilitates project within the practice		

 IRB Application – TRANSLATE CKD Trial. Property of the Authors. All rights reserved.

REVISED TRANSLATE scoring tool 3-11-2015.xlsx

	S5	Site coordinator meets deadlines for deliverables		
L	L0	**Local clinician champion**	0	
	L1	Clinician champion identified		
	L2	Clinician champion meets regularly with study personnel		
	L3	Clinician champion is responsive to the needs of study team		
	L4	Clinician champion engages other providers regarding CKD project		
	L5	Clinician champion meets deadlines for deliverables		
A	F0	**Audit and Feedback**	0	
	F1	Regularly reviews CKD performance reports with study team		
	F2	Routinely disseminates CKD performance reports t/o practice		
	F3	Changes workflow in response to performance reporting		
	F4	Has designated staff for population health management role		
	F5	Develops capacity to create in-house performance reports		
T	T0	**Team Approach**	0	
	T1	Routine care team meetings about individual patient care		
	T2	Working to the highest level of license (not below)		
	T3	PCMH certification status		
	T4	Care teams are actively engaged in study		
	T5	Integrates CKD patient self-management support into workflows		
E	E0	**Education**	0	
	E1	Participates in regular CKD academic mentoring (study/other)		
	E2	Routinely uses education materials/tools provided by the study		
	E3	Engages in CKD study collaborative learning opportunities		
	E4	Incorporates CKD study into in-house training		
	E5	Trains practice on population health management (study/other)		
		**TOTAL**	0	
