# Supplementary material for: Effect of 2 Clinical Decision Support Strategies on Chronic Kidney Disease Outcomes in Primary Care: A Cluster Randomized Trial
Source: JAMA Netw Open. 2018 Oct 26;1(6):e183377. doi: 10.1001/jamanetworkopen.2018.3377 (PMC6324427; doi:10.1001/jamanetworkopen.2018.3377)
Supplement: Supplement 2. — eTable 1. TRANSLATE Elements as Implemented eReferences eTable 2. List of EHRs eTable 3. Intent to Treat Analysis Of Stage 3 and Stage 4 Patients From the Final Cohort Of Practices. Systolic BP Change Over Time: Intervention vs Randomized Controls eTable 4. Other Secondary Outcomes: Intervention vs Randomized Controls eFigure 1. Data Sources for the Creation of the Point of Care Reminder eFigure 2. Map of Practice Locations [file jamanetwopen-1-e183377-s002.pdf]

Supplementary Online Content

Carroll JK, Pulver G, Dickinson LM, et al. Effect of 2 clinical decision support strategies on chronic kidney disease outcomes in primary care: a cluster randomized trial. *JAMA Netw Open*. 2018;1(6):e183377. doi:10.1001/jamanetworkopen.2018.3377

eTable 1. TRANSLATE Elements as Implemented

eReferences

eTable 2. List of EHRs

eTable 3. Intent to Treat Analysis Of Stage 3 and Stage 4 Patients From the Final Cohort Of Practices. Systolic BP Change Over Time: Intervention vs Randomized Controls

eTable 4. Other Secondary Outcomes: Intervention vs Randomized Controls

eFigure 1. Data Sources for the Creation of the Point of Care Reminder

eFigure 2. Map of Practice Locations

This supplementary material has been provided by the authors to give readers additional information about their work.

© 2018 Carroll JK et al. *JAMA Network Open.*

eTable 1. TRANSLATE Elements as Implemented

TRANSLATE Element	Operationalized As	CDS	CDS+PF
Set your **T**arget	Common targets for chronic kidney disease (CKD) detection and treatment were created and communicated via a CKD tracking tool that was provided to all sites. The key performance measures and their targets were control of blood pressure, glucose, low density lipoprotein (LDL), use of angiotensin converting enzyme inhibitor (ACEi)/ angiotensin receptor blocker (ARB), referral to a nephrologist, smoking cessation and avoidance of nonsteroidal anti-inflammatory drugs (NSAID) or Cyclooxygenase-2 (Cox-2) medications.	✓	✓
Use point of care **R**eminder systems	A point-of-care decision support tool prompted users to implement the key evidence-based recommendations that were the focus of the study.	✓	✓
Get **A**dministrative buy-in	Study personnel obtained consent from each practice and worked with all physicians in the practices.	✓	✓
**N**etwork Information systems utilizing registries	A system-level registry reports across all practices. The registry was provided through practices' electronic health records (EHRs) and interaction with DARTNet and CINA, companies that collect, standardize, and synthesize data from multiple EHR vendors.	✓	✓
**S**ite coordination	A site coordinator at each practice assembled a quality improvement team that met monthly to review performance data regarding CKD. The site coordinator created local accountability for completion of the study, and also worked with the clinicians and practice staff to implement workflow changes such as pre-visit planning, standing orders, and patient education materials to improve disease management.		✓
**L**ocal Physician Champion	A clinician leader in each practice supported the site coordinator and the quality improvement team. This clinician was in contact with an academic mentor regarding clinical questions about CKD and participated in learning collaboratives with the site coordinator.		✓

© 2018 Carroll JK et al. *JAMA Network Open.*

TRANSLATE Element	Operationalized As	CDS	CDS+PF
**A**udit and feedback	Audit and Feedback reports were generated at the practice, individual provider, and patient level through CINA regarding the seven performance measures (blood pressure, hemoglobin A1c (HbA1c), LDL, use of ACE/ARB, referral to a nephrologist, smoking cessation and avoidance of NSAID or Cox-2). Reports were reviewed quarterly with the practice facilitator by videoconference.		✓
**T**eam approach	The local physician champion, site coordinator and nursing, front office, and administrative staff met monthly to review progress of the CKD project. Workflow changes were recommended and tested.		✓
**E**ducation	An Educational program using academic detailing,[1] practice facilitation,[2] and video conferencing was implemented to support the practices' efforts. All practices were assigned an academic practice mentor (Drs. Fox or Vassalotti). The mentors were available to the office physician champion and practice coordinator to answer questions. The academic mentors reviewed the practice's data and participated in quarterly videoconferences with either the study coordinator or the lead clinician to review progress on the project. The practice facilitators were available remotely and completed facilitation training. They provided support to build the capacity of the practice to continuously evaluate and implement sustained improvements in evidence-based CKD care. Facilitators also participated in regular study team meetings, data collection, and communication with participating sites. Additional description of the facilitators' role has been published.[3]		✓

✓ = implemented in this study group
CDS = computerized decision support
CDS+PF = computerized decision support plus practice facilitation

References

1. Soumerai SB, Avorn J. Principles of educational outreach ('academic detailing') to improve clinical decision making. *JAMA : the journal of the American Medical Association.* 1990;263(4):549-556.
2. Nagykaldi Z, Mold JW, Robinson A, Niebauer L, Ford A. Practice Facilitators and Practice-based Research Networks. *Journal of the American Board of Family Medicine: JABFM.* 2006;19(5):506-510.
3. Fox CH, Vest BM, Kahn LS, et al. Improving evidence-based primary care for chronic kidney disease: study protocol for a cluster randomized control trial for translating evidence into practice (TRANSLATE CKD). *Implementation science : IS.* 2013;8:88.

© 2018 Carroll JK et al. *JAMA Network Open.*

eTable 2. List of EHRs

EHR Name	Count
EMDs	16
i2i	8
Allscripts	5
Medinformatix	1
Centricity	1
Medent	6
HealthMetrics	5
**Total**	**42**

© 2018 Carroll JK et al. *JAMA Network Open.*

eTable 3. Intent to Treat analysis of stage 3 and stage 4 patients from the final cohort of practices. Systolic BP change over time: Intervention vs randomized controls

Systolic BP	Intent to Treat		Propensity Adjusted	
Variable	Adj models Coef (SE)	p-value	Adj models Coef (SE)	p-value
Intercept	128.65 (1.76)	----	130.09 (1.94)	----
Age	.11 (.01)	<.001	.11 (.01)	<.001
Gender				
Female (ref)				
Male	-1.21 (.26)	<.001	-1.25 (.26)	<.001
Current smoker	-.53 (.47)	.26	-.56 (.47)	.23
Diabetes	1.07 (.38)	.005	1.03 (.38)	.007
ACEi/ARB	2.91 (.27)	<.001	2.63 (.32)	<.001
Stage 4	.77 (.31)	.01	.78 (.31)	.01
CKD diagnosis	-.17 (.39)	.66	-.75 (.51)	.14
NSAIDs	-.20 (.29)	.49	-.25 (.29)	.39
LDL<100	-1.59 (.26)	<.001	-1.56 (.26)	<.001
HbA1c				
Less than 7 (ref)	----		----	
7 or greater	1.94 (.37)	<.001	1.97 (.37)	<.001
Not done	-.06 (.37)	.87	-.09 (.37)	.81
Intervention vs controls (at baseline)	2.51 (2.05)	.22	2.82 (2.05)	.17
Systolic blood pressure change per year in controls (slope)	.29 (.30)	.34	.29 (.30)	.33
Difference in blood pressure slope for intervention patients	-.10 (.35)	.77	-.10 (.35)	.77

© 2018 Carroll JK et al. *JAMA Network Open.*

ACEi = angiotensin converting enzyme inhibitor

ARB = angiotensin receptor blocker

CKD = Chronic Kidney Disease

HbA1c = hemoglobin A1c

LDL = low density lipoprotein

NSAID = nonsteroidal anti-inflammatory drugs

SE = standard error

© 2018 Carroll JK et al. *JAMA Network Open.*

eTable 4. Other secondary outcomes: Intervention vs randomized controls

	Use of NSAIDS N=6699				Use of ACEi/ARB N=6699				CKD diagnosis* N=5331			
	Intent to Treat		Propensity Adjusted		Intent to Treat		Propensity Adjusted		Intent to Treat		Propensity Adjusted	
Variable	Adj models Coef (SE)	p-value	Adj models Coef (SE)	p-value	Adj models Coef (SE)	p-value	Adj models Coef (SE)	p-value	Adj models Coef (SE)	p-value	Adj models Coef (SE)	p-value
Intercept	-1.64(.57)	----	-1.30 (.66)	----	0.19 (.55)	---	.62 (.63)	----	-1.09 (.67)	---	-.47 (.60)	----
Age	-.004 (.006)	.50	-.004 (.006)	.51	.002 (.007)	.80	.002 (.007)	.73	-.005 (.01)	.59	-.005 (.01)	.64
Gender												
Female (ref)	-----		----		---				---		---	
Male	-.21 (.10)	.03	-.21 (.09)	.02	-.101(.03)	<.001	-.13 (.02)	<.001	.41 (.22)	.06	.40 (.23)	.08
Current smoker	-.16 (.05)	.002	-.17 (.05)	<.001	-.07 (.08)	.38	-.11 (.06)	.08	-.04 (.06)	.46	-.08 (.07)	.26
Diabetes	-.21(.06)	<.001	-.23 (.06)	<.001	.45 (.12)	<.001	.41 (.12)	001<.	.37 (.14)	.009	.33 (.12)	.008
Stage 4	-.49 (.10)	<.001	-.49 (.11)	<.001	-.15(.07)	.04	-.17 (.06)	.003	1.01 (.21)	<.001	1.02 (.21)	<.001
CKD dx at baseline	-.47 (.20)	.02	-.63 (.27)	.02	.27 (.21)	.20	.24 (.22)	.28	----	----	----	----
BP≤140/90	.18 (.11)	.08	.19 (.11)	.07	-.02 (.13)	.85	-.01 (.13)	.94	-.13 (.12)	.29	-.11 (.12)	.35
LDL<100	-.08(.05)	.12	-.08 (.05)	.14	0.05(.05)	.29	.05 (.05)	.28	.16 (.08)	.07	.16 (.08)	.03
HbA1c less than 7 (ref)	---	---	---		---	---	---		---	---	----	----
7 or greater	-13 (.12)	.27	-.13 (.12)	.28	.19 (.05)	<.001	.19 (.05)	<.001	.24 (.06)	<.001	.25 (.06)	<.001
Not done	-.19 (.09)	.04	-.19 (.09)	.03	-.12 (.11)	.26	-.12 (.11)	.26	-.14 (.15)	.35	-.15 (.14)	.29
Intervention (vs controls)	0.42 (.34)	.22	.51 (.38)	.18	-.52(.47)	.27	-.30 (.53)	.57	-.04 (.06)	.46	-.07 (.29)	.81

*among those who did not have a CKD dx at baseline

CKD = Chronic Kidney Disease

HbA1c = hemoglobin A1c

NSAID = nonsteroidal anti-inflammatory drugs

ACEi = angiotensin converting enzyme inhibitor

ARB = angiotensin receptor blocker

© 2018 Carroll JK et al. *JAMA Network Open.*

© 2018 Carroll JK et al. *JAMA Network Open.*

eFigure 1. Data sources for the creation of the point of care reminder

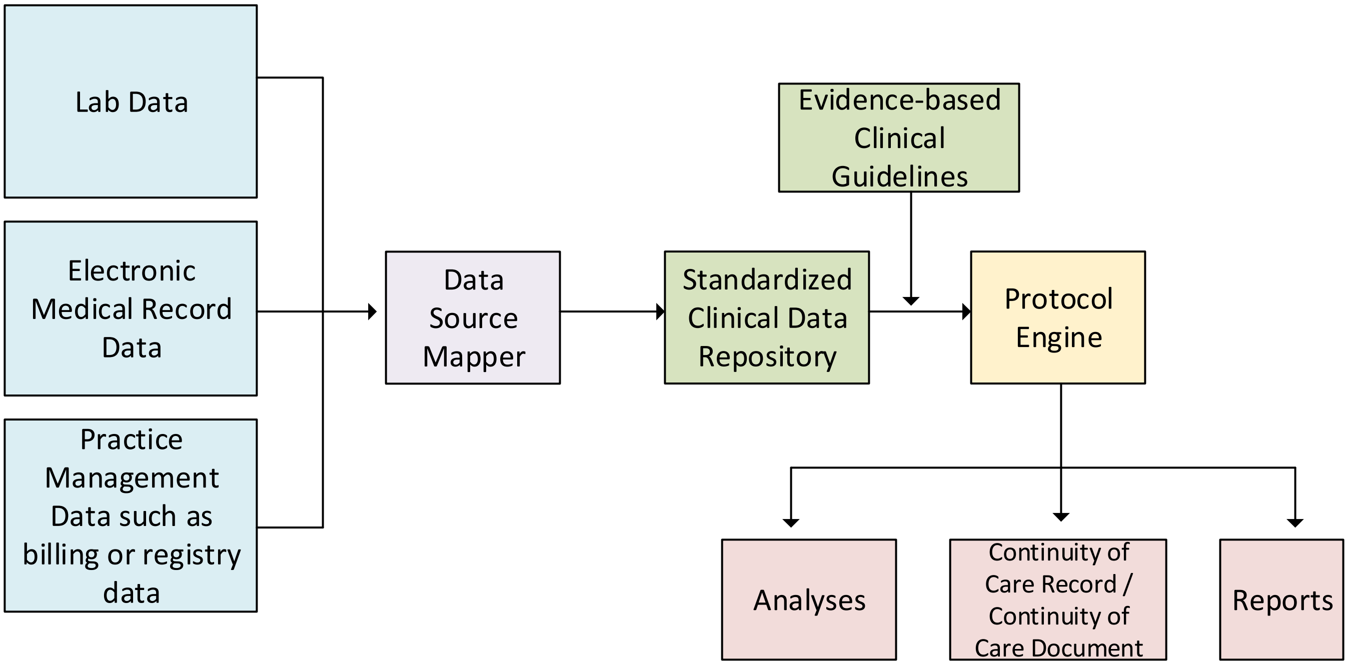

© 2018 Carroll JK et al. *JAMA Network Open.*

eFigure 2: Map of practice locations

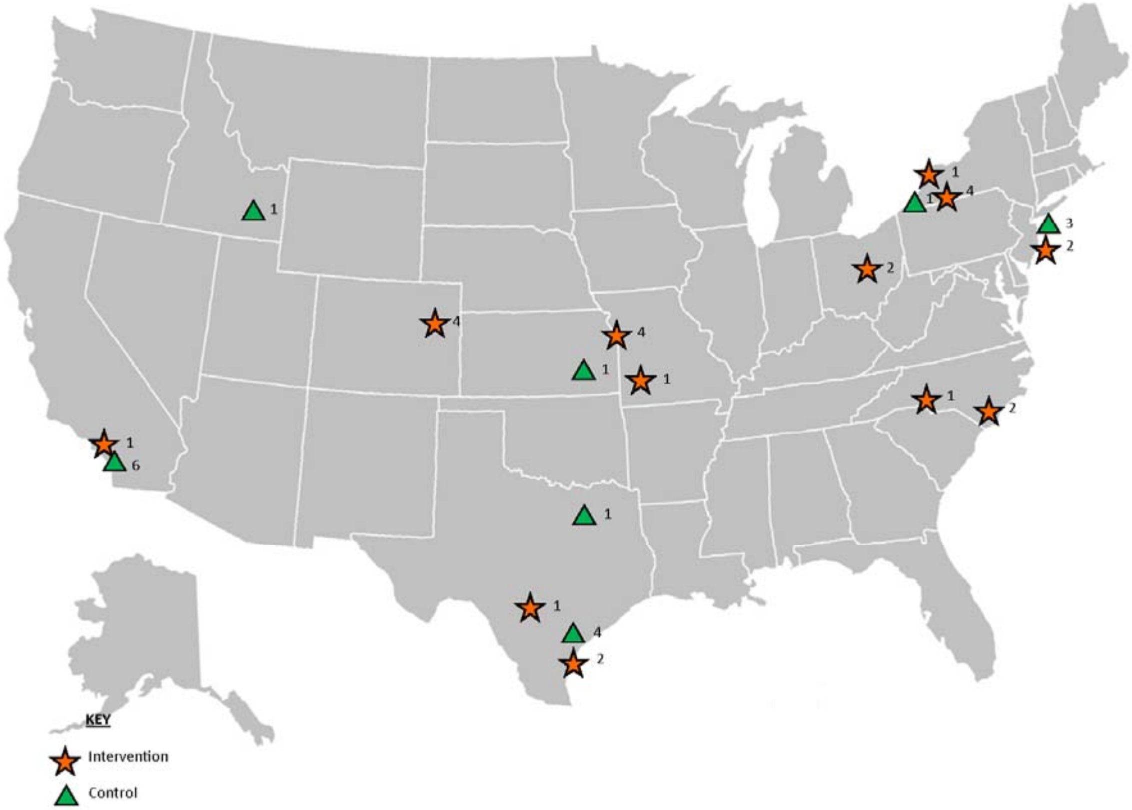

© 2018 Carroll JK et al. *JAMA Network Open.*
