# Supplementary material for: Effect of 2 Clinical Decision Support Strategies on Chronic Kidney Disease Outcomes in Primary Care: A Cluster Randomized Trial
Source: JAMA Netw Open. 2018 Oct 26;1(6):e183377. doi: 10.1001/jamanetworkopen.2018.3377 (PMC6324427; doi:10.1001/jamanetworkopen.2018.3377)
Supplement: Supplement 3. — Data Sharing Statement [file jamanetwopen-1-e183377-s003.pdf]

Data Sharing Statement

Carroll. Effect of 2 Clinical Decision Support Strategies on Chronic Kidney Disease Outcomes in Primary Care. *JAMA Network Open*. Published October 26, 2018. 10.1001/jamanetworkopen.2018.3377

Data
Data available: Yes
Data types: Deidentified participant data, Data dictionary, Other (please specify)
Additional Information: calculated values for some variables
How to access data: Rachel Bryan Kent, Director of Client Services, DARTNet Institute Rachel.Kent@dartnet.info www.dartnet.info
When available: With publication

Supporting Documents
Document types: None

Additional Information
Who can access the data: Researchers whose proposed use of the data has been approved.
Types of analyses: Any purpose that is appropriate to the data on hand.
Mechanisms of data availability: With a signed data access agreement and review of the proposal to be sure the data can support the question(s).
Any additional restrictions: Data cannot be used to attempt to re-identify any sites or patients and contact them in any manner. These data contain PHI - dates of service. The data would need to be fully de-identified prior to being supplied to other people. Funds to offset that work would be required, but we are fine with sharing once fully de-identified in a manner that works for the research question. The de-identification approach could vary depending on the research question.
